# Nutrient effects on drought responses vary across common temperate grassland species

**DOI:** 10.1007/s00442-023-05370-5

**Published:** 2023-05-05

**Authors:** Carola Kiene, Eun-Young Jung, Bettina M. J. Engelbrecht

**Affiliations:** 1 Functional and Tropical Plant Ecology, Bayreuth Centre of Ecology and Environmental Research (BayCEER), University of Bayreuth, 95440 Bayreuth, Germany; 2 Smithsonian Tropical Research Institute, Apartado, 0843-03092 Balboa, Ancon Republic of Panama

**Keywords:** Drought tolerance, Global change, Nitrogen, Phosphorus, Performance ranking

## Abstract

Drought and nutrient input are two main global change drivers that threaten ecosystem function and services. Resolving the interactive effects of human-induced stressors on individual species is necessary to improve our understanding of community and ecosystem responses. This study comparatively assessed how different nutrient conditions affect whole-plant drought responses across 13 common temperate grassland species. We conducted a fully factorial drought-fertilization experiment to examine the effect of nutrient addition [nitrogen (N), phosphorus (P), and combined NP] on species' drought survival, and on drought resistance of growth as well as drought legacy effects. Drought had an overall negative effect on survival and growth, and the adverse drought effects extended into the next growing season. Neither drought resistance nor legacy effects exhibited an overall effect of nutrients. Instead, both the size and the direction of the effects differed strongly among species and between nutrient conditions. Consistently, species performance ranking under drought changed with nitrogen availability. The idiosyncratic responses of species to drought under different nutrient conditions may underlie the seemingly contradicting effects of drought in studies on grassland composition and productivity along nutrient and land-use gradients—ranging from amplifying to dampening. Differential species’ responses to combinations of nutrients and drought, as observed in our study, complicate predictions of community and ecosystem responses to climate and land-use changes. Moreover, they highlight the urgent need for an improved understanding of the mechanisms that render species more or less vulnerable to drought under different nutrients.

**Supplementary Information:**

The online version contains supplementary material available at 10.1007/s00442-023-05370-5.

## Introduction

Drought and increasing nutrient loads are two main global change drivers and threaten ecosystem function and services (Sala et al. 2000). The frequency and intensity of drought events are projected to increase with global climate change in temperate grassland ecosystems (IPCC 2014). Simultaneously, they are exposed to increased nutrient availability directly through fertilizer application or indirectly through atmospheric nitrogen deposition. Understanding the joint effects of drought and nutrients in temperate grasslands is especially important, because they are among the most widespread biomes, exhibit high species richness, and provide essential and economically relevant ecosystem services (Gibson 2009; Wilson et al. 2012). However, how drought and nutrients in combination affect grassland systems remains largely unresolved.

Individually, drought and nutrients have contrasting effects on plant performance. Periods of low soil water availability, i.e., drought (Gilbert and Medina 2016), often lead to decreases in plant growth and survival, as well as declines in community diversity and ecosystem productivity (Tilman and El Haddi 1992; Fay et al. 2003; Knapp et al. 2015). Such effects can occur during the drought, but drought effects can also persist after the drought has ended (drought legacy effects, Reichmann and Sala 2014; Vilonen et al. 2022). On the other hand, nutrient availability, especially nitrogen, increases plant growth and ecosystem productivity and leads to decreased diversity (Bobbink et al. 2010; Socher et al. 2012; Soons et al. 2017). Studies considering both factors indicate pervasive interactive effects of nutrient and water availability. Amplifying as well as dampening effects of nutrients on drought responses have been found at both the species and community level (e.g., Carlsson et al. 2017; Hofer et al. 2017; Kübert et al. 2019; Bharath et al. 2020; Van Sundert et al. 2021; Meng et al. 2021). Drought resistance, i.e., the ability to minimize the adverse effects of low water availability during drought, has been suggested to be low for species in high nutrient sites. Those species often exhibit an acquisitive resource-use strategy, with high resource capture, fast tissue turnover, and high growth rates. This strategy should render species sensitive to drought, leading to an amplifying effect of nutrients on drought responses. On the other hand, a conservative resource-use strategy should allow species associated with low nutrient sites to cope with both low nutrients and low water availability (e.g., Grime et al. 2000; Reich 2014; Eskelinen and Harrison 2015). Additionally, phenotypic changes of trait expression in response to nutrients, such as reduced root-shoot ratios, which are often associated with decreased water uptake capacity and higher transpiration, may lead to amplification of negative drought effects under high nutrients (e.g., Friedrich et al. 2012; Wang et al. 2018; Kübert et al. 2019; Bharath et al. 2020; Meng et al. 2021). Negative effects of high nutrient availability on drought responses have therefore been widely assumed (e.g., Bobbink et al. 2010; Meyer-Grünefeldt et al. 2013; Reich 2014). However, high nutrients have also been shown to dampen negative drought effects, i.e., increase plant drought performance (Carlsson et al. 2017; Hofer et al. 2017). Improved plant vigor due to release from nutrient limitation, increased photosynthesis, deeper roots, or resource storage under high nutrient availability prior to drought may dampen negative drought effects or lead to positive legacy effects of drought (Karlowsky et al. 2018).

Interactions between drought and nutrients may indeed vary in direction and size among species and plant life forms. Differences in species’ resource-use strategy, their nutrient limitation, and/or differences in traits related to drought resistance and their phenotypic responses to nutrients may underlie such variation (Goldstein et al. 2013; Hofer et al. 2017; Van Sundert et al. 2021). Indeed, several studies suggest different effects of nutrients on drought performance in different life forms, based on differences in root traits and/or resource acquisition strategy. Finer, shallower roots and a more acquisitive strategy may underlie observations of stronger decreases of drought resistance in response to nutrient in grasses than in forbs (Friedrich et al. 2012; Kübert et al. 2019; Van Sundert et al. 2021). Other studies have shown that nitrogen addition released nutrient limitation and enhanced drought resistance in grasses and non-legume forbs, but not in nitrogen-fixing legumes (Hofer et al. 2017; Carlsson et al. 2017). The contribution of drought resistance vs recovery for drought legacy effects can additionally differ among species and life forms (Hofer et al. 2016; Karlowsky et al. 2018). Differential performance responses of species or life forms may impede an overall consistent positive or negative impact of nutrients on drought responses across species and communities. If nutrients have differential effects on drought responses across species, this may lead to changes of species drought performance ranking across gradients of nutrient availability. Altered species hierarchies can thus affect competitive interactions and community composition. To improve our understanding, we need to gain insights into the combined effects of nutrient availability and drought on plant performance (i.e., growth and survival) and how these effects vary across species.

Toward this aim, experimental approaches are necessary, which comparatively assess species' whole-plant performance responses, which integrate responses at various levels of organization (genetic, physiological, organ in Blum 1996), and are most directly relevant for fitness and productivity. However, comparative studies across species remain scarce, especially with multiple environmental factors. So far, a few studies that explicitly focused on comparing whole-plant performance responses across numerous species considered either drought or nutrients (e.g., drought in Engelbrecht et al. 2007; Jung et al. 2020, nutrients in Wilson and Tilman 1991; Zhang et al. 2015). Experimental assessments of the effects of both water and nutrient availability on performance responses were restricted to single or few species and/or to the addition of only one nutrient (e.g., Friedrich et al. 2012; Chieppa et al. 2019). The lack of comparative studies considering combined effects of drought and nutrients across species hampers the understanding and predictions of the role nutrients play for species and community drought responses, including in temperate grasslands.

In this study, we comparatively quantified whole-plant drought responses of 13 common temperate grassland species under different nutrient regimes in a common garden experiment. We assessed drought resistance (i.e., the ability to maintain growth during drought), drought legacy effects (i.e., drought effects on growth persisting after the drought has subsided, sensu Vilonen et al. 2022, compare Glossary S1), and drought survival responses in a fully factorial experimental design with four nutrient treatments combined with two moisture treatments (drought and irrigation treatment). The approach allowed us to directly compare under different nutrient conditions the performance of drought stressed plants with plants that did not experience drought stress (irrigation treatment) during and after the experimental drought period. We hypothesized that a combination of drought and nutrient addition can have two possible outcomes for whole-plant drought survival, drought resistance, and drought legacy effects: (a) nutrient addition has a consistent negative effect across species, or (b) the size and/or direction of nutrient effects varies across species (i.e., drought and nutrient effects interact). Such variation may be related to species habitat association, resource-use strategies, nutrient limitation, or root allocation. We also tested if species performance ranks under drought change across nutrient conditions and if the nutrient effect on drought responses differs among plant life forms.

## Materials and methods

### Study site and study species

The experiment was conducted at the Ecological Botanical Garden of the University of Bayreuth, Germany (49°55′19′′ N, 11°34′55′′ E) in 2017–2019. The region has a temperate climate with a mean annual temperature of 8 °C and mean annual precipitation of 760 mm (see Table S1, Methods S2 for further details). The study was conducted on 13 common temperate, perennial grassland species (Table 1) that are common in extensively managed grasslands in Germany (Socher et al. 2012) and included a wide range of moisture and nutrient associations (*F *values 3–7, *N *values 1–8, based on Ellenberger indicator values Ellenberg et al. 1992). The study species consisted of six grasses and seven forbs (among them three legumes), and all had C_3_ photosynthetic pathway. Three additional species were planted into the experiment but consequently excluded from analyses of drought performance because of low survival in the establishment period (*Medicago lupulina* and *Bromus hordeaceus*) or seed contamination with another species (*Trisetum flavescens*). They were, however, considered in the models as 'neighbor biomass'.

**Table 1:** List of the study species

Species	Code	Family	Plant life form
*Achillea millefolium* L	ACHI MI	Asteraceae	forb
*Briza media* L	BRIZ ME	Poaceae	grass
*Cerastium holosteoides* Fr	CERA HO	Caryophyllaceae	forb
*Dactylis glomerata* L. ssp. *glomerata*	DACT GL	Poaceae	grass
*Festuca ovina*L. agg	FEST OV	Poaceae	grass
*Helictotrichon pubescens (Huds.) Pilg. ssp. Pubescens*	HELI PU	Poaceae	grass
*Lotus corniculatus* L	LOTU CO	Fabaceae	legume
*Phleum pratense* L	PHLE PR	Poaceae	grass
*Poa trivialis* L. ssp. *trivialis*	POA TR	Poaceae	grass
*Ranunculus bulbosus* L. ssp. *bulbosus*	RANU BU	Ranunculaceae	forb
*Taraxacum sect. Ruderalia,* *Kirschner, H. Øllg. & Štěpánek*	TARA RU	Asteraceae	forb
*Trifolium repens* L	TRIF RE	Fabaceae	legume
*Vicia cracca* L	VICI CR	Fabaceae	legume

Given are the species name, code, family, and plant life form (grasses and forbs, including legumes)

### Experimental design and timeline

Seedlings were germinated in the greenhouse and transplanted to large plastic boxes (120 × 100 × 100 cm; *L* × *W* × *H*) in a common garden in June 2017. Plants were grown under four different nutrient conditions: addition of nitrogen (N), or phosphorus (P) individually, combined addition (NP), and unfertilized control (C), with 18 boxes for each nutrient treatment. One individual per species was planted in each box. Plants were grown under these nutrient conditions with ample water supply for about 1 year (establishment period) to allow for acclimation and trait expression under the different nutrient treatments. Then, two soil moisture treatments were applied: drought and irrigated controls. To that end, all boxes were covered with transparent rainout shelters and a drought treatment was implemented in each nutrient treatment for 8 weeks (56 days, drought period) to half of the boxes (irrigation was discontinued). The other half of the boxes served as control for the drought treatment and remained irrigated throughout (irrigation treatment). Each nutrient × moisture treatment was thus replicated nine times (for a schematic representation of the block design and the experimental timeline, see Figs. S1, S2, see Glossary S1 for summary terms). To assess potential effects persisting after the drought ends (legacy effects, compare Vilonen et al. 2022), the droughted boxes were re-irrigated after termination of the moisture treatments, rainout shelters were removed, and all plants were irrigated and exposed to natural weather conditions until the following growing period (post-drought period, until May 2019). Growing the plants jointly in large plastic boxes allowed for slow progressive soil drying and extended and deep root development. Furthermore, this approach avoided problems with comparing drought effects across species or growing conditions for plants in individual pots (Comita and Engelbrecht 2014). For plant performance assessments, see below.

### Nutrient and moisture conditions

Plants were grown on a baseline soil substrate (sandy loam) with relatively low nutrient concentrations. Nutrient conditions were chosen to mimic typical fertilization levels in agricultural grasslands in Germany (Blüthgen et al. 2012; Vogt et al. 2019) and to be comparable to other fertilization studies in temperate grasslands (e.g., Borer et al. 2014; Weisser et al. 2017). The N-addition treatment received the equivalent of 100 kg N ha^−1^ year^−1^, P addition the equivalent of 50 kg P ha^−1^ year^−1^, and the NP treatment a combination of both. The fertilization was strongly reflected in leaf nutrient contents, despite only minor effects on soil nutrient contents (Methods S2, Tables S2, S3).

During the drought period, soil water potentials in the irrigation treatment stayed above—0.16 MPa (upper 20 cm), while in the drought treatment, soil water potentials reached below—1.5 MPa (considered permanent wilting point) after 11.73 ± 0.89 days (mean ± SD**)**. The drought condition in the experiment was intense, as indicated by soil water potentials below—3.3 MPa at the end of the 56 days of drought treatment (Fig. S3) and the fact that 42 consecutive days without rain are considered an extreme 1000-year meteorological drought event in the study area (Jentsch et al. 2011). Light transmittance of the rainout shelters was 86% (assessed with AP4, Delta-T, Cambridge), and air temperature and relative air humidity did not differ between treatments (for details, see Methods S2).

### Assessment of plant performance

We assessed performance based on growth and survival for all individual plants. Additionally, we visually classified drought damage weekly for all droughted plants during the drought period (for classification categories, see Table S4). Growth rates were assessed in both moisture treatments over the drought period (GR_drought_, GR_irrigated_) and over the post-drought period (GR_post-drought_, GR_post-irrigated_) as aboveground biomass increase per time, based on the biomass developed by each individual between two harvests. Growth rates were then used to calculate drought resistance and drought legacy effects for each species (see below). Survival was monitored after the post-drought phase. We did not monitor survival directly after the drought phase to avoid erroneously considering individuals as dead that experienced total loss aboveground biomass during the drought phase, but resprouted from surviving underground meristems after the drought (compare Jung et al. 2020; for details, see Fig. S2, Glossary S1 and Methods S2).

To explicitly test for each species if the response to drought (resistance, legacy effects, and survival) differed among nutrient conditions, we calculated drought response ratios for each species as the performance in the drought relative to the irrigation treatment for each nutrient treatment. Specifically, drought resistance (*D*_resist_) of growth was calculated for each individual plant in the drought treatment (GR_drought_), relative to the mean growth of the species in the irrigation treatment $\left( {\mathop {\overline{{{\text{GR}}}} }\nolimits_{{{\text{irrigated}}}} } \right)$ during the drought phase in the respective nutrient condition:$\mathop D\nolimits_{{{\text{resist}}}} = \frac{{\mathop {{\text{GR}}}\nolimits_{{{\text{drought}}}} }}{{\mathop {\overline{{{\text{GR}}}} }\nolimits_{{{\text{irrigated}}}} }}$. Potential drought legacy effects (D_legacy_) on growth in the post-drought periods were analogously determined as the ratio of growth of each formerly droughted individual (GR_post-drought_) relative to the mean growth of the irrigation plants in the post-drought phase $\left( {\mathop {\overline{GR} }\nolimits_{post - irrigated} } \right)$, separately for each species and nutrient condition) as $\mathop D\nolimits_{{{\text{legacy}}}} = \frac{{\mathop {{\text{GR}}}\nolimits_{{\text{post - drought }}} }}{{\mathop {\overline{{{\text{GR}}}} }\nolimits_{{\text{post - irrigated}}} }}$. Drought survival response (*D*_surv_) was determined at the species level for each nutrient condition, as whole-plant survival in drought (S_drought_) relative to the irrigation treatment (*S*_irrigated_) at the end of the experiment (after the post-drought phase) as $\mathop D\nolimits_{{{\text{surv}}}} = \frac{{\mathop S\nolimits_{{{\text{drought}}}} }}{{\mathop S\nolimits_{{{\text{irrigated}}}} }}$, where S was quantified as the percentage of surviving individuals in each species relative to the initial number of individuals (across boxes in each respective treatment). For all three parameters, drought response values < 1 thus indicate lower performance in the (former) drought relative to the irrigation treatment (with lower values indicating larger negative effects of drought), and response values > 1 indicate higher performance of plants that experience(d) drought conditions. Note that our index of drought legacy effects compares under the same environmental conditions (moisture, nutrient treatment, but also light, temperature, etc.) the growth of individuals that have experienced experimental drought stress to those that have not experienced drought stress throughout the study period. It thus directly reflects the effects of former drought on growth (of the different species and under different nutrient conditions) after the drought has subsided (compare Vilonen et al. 2022).

### Statistical analyses

#### Effects of drought and nutrients on plant performance

We first tested the effects of nutrient conditions (N, P), drought (D), and all possible interactions across species on survival, growth rates during the drought period, and growth rates in the post-drought period. We fitted separate models for each performance parameter, using binomial generalized linear mixed-effects models (GLMM using the lme4 package, Bates et al. 2015) for survival and linear mixed models (LMM) for growth rates, respectively. Species were included as a random effect in these models. All analyses of growth in the main text refer to surviving individuals, contributing to future population dynamics, except for *Ranunculus bulbosus* in the NP, drought treatment, where no individuals survived. Here, all individuals were considered.

We then tested the effect of N addition (N), P addition (P), and their interactions on drought resistance and drought legacy effects (*D*_resist_ and *D*_legacy_) across all species, including species (Sp; 13 species) or including plant life forms (Lf: non-leguminous forbs, legumes, and grasses), respectively, and their interactions with the nutrient treatments. We used separate LMMs for each performance parameter (*D*_resist_ and *D*_legacy_) and each level (across species, Lf, Sp). Effects of nutrient conditions on drought survival responses (*D*_surv_) were tested across all species and in interaction with life forms. Species were included as a random effect in models across species and contained Lf as an explanatory variable. To account for the blocked design in the common garden experiment and possible effects of neighbors on target plants, we included block and the pooled biomass of directly neighboring plants as random effects. The significance of the fixed factors in the GLMMs and LMMs was calculated with a Wald test on the full model using the car package (Fox and Weisberg 2019). The significance of nutrient effects (N, P) within each species or life form, respectively, was assessed with a post hoc test for multiple comparisons among nutrient conditions using the holm method with the emmeans package (Lenth et al. 2018). The significance of random term was assessed with a likelihood-ratio test between a model with and one without the term of interest (Zuur et al. 2009).

All models were tested for normal distribution (quantile–quantile plots and Shapiro–Wilk test) and homoscedasticity (residual plots and Levene test). If these criteria were not met, models were corrected by including a variance function (nlme package; Pinheiro et al. 2019) or transformation. Marginal *R*^2^ (fixed effects only) and conditional *R*^2^ (random and fixed effects) of the regression models are provided.

#### Parameters associated with variation of nutrient effects on drought responses

Using linear models, we assessed if variation of species drought responses (*D*_surv_, *D*_resist_, *D*_legacy_) with nutrient addition is related to their habitat association (based on Ellenberg indicator values for moisture and nitrogen, *F* and *N* values, Ellenberg et al. 1992), resource-use strategy (based on specific leaf area), root allocation (based on root mass ratio under full recourse availability; irrigated, NP), or nutrient limitation [assessed as the difference in growth rate (ΔGR) between fertilized and unfertilized plants, separately for each nutrient addition (N, P, NP) under drought and irrigated conditions]. For details, see Methods S2.

#### Effects of drought and nutrient conditions on species performance ranks

To test for changes in performance ranks across nutrient conditions within each moisture treatment, we calculated Spearman rank correlation coefficients between pairwise combinations of nutrient conditions for each of the performance parameters (survival, growth rates during the drought period, and growth rates in the post-drought period). A significant positive relation indicates that the species rank hierarchy was maintained, and a negative relationship that the species rank hierarchy was reversed. A lack of significant correlation suggests that species hierarchical status changed idiosyncratically between nutrient treatments (compare, e.g., Kitajima and Bolker 2003).

All statistical analyses were performed in R version 3.6.2 (R Core Team 2019). A significance level of *P* ≤ 0.05 was used throughout.

## Results

### Effects of nutrients and drought on survival

**Fig. 1:**
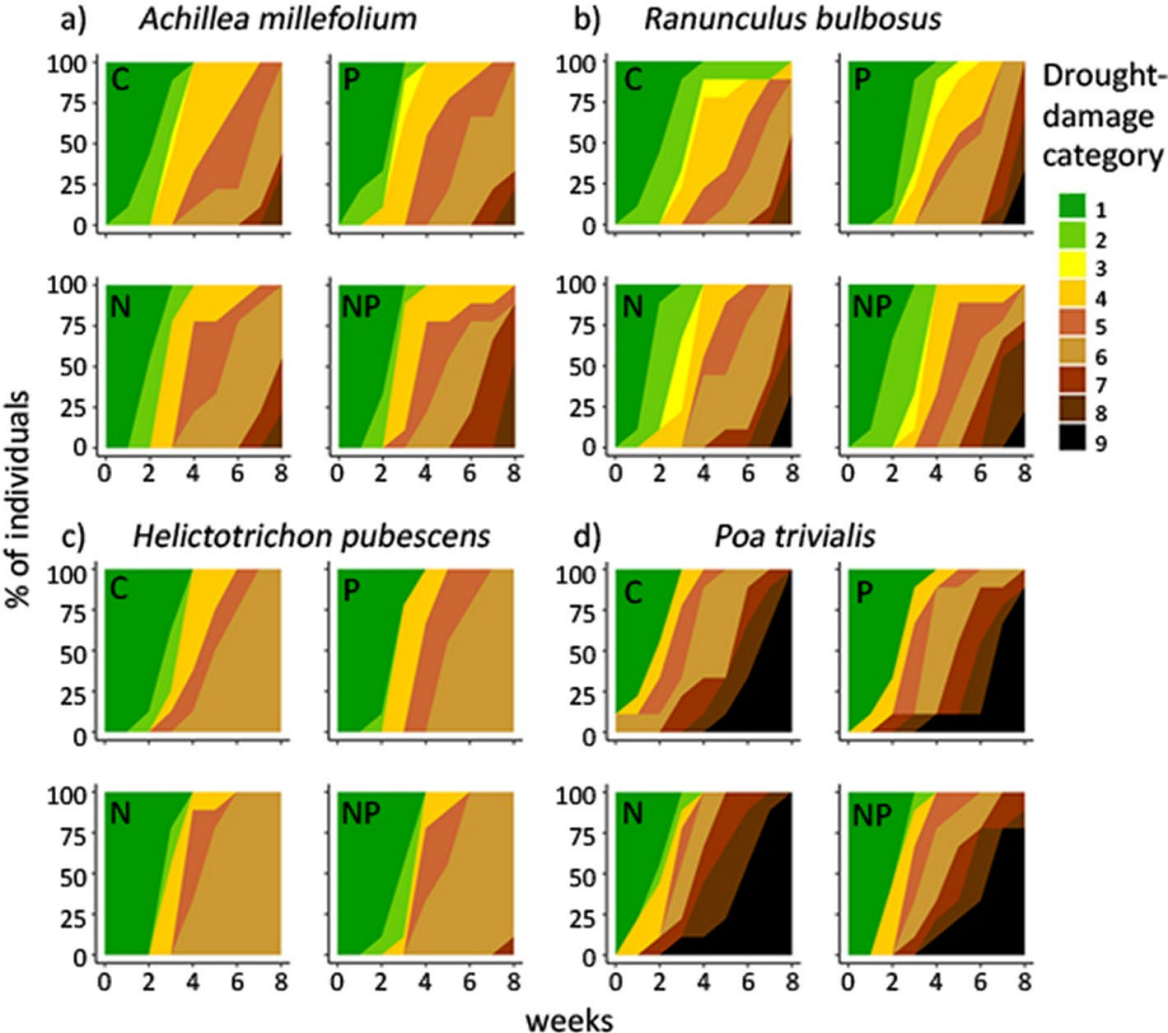
Progression of visual drought damage under four different nutrient conditions over 8 weeks of drought. Shown is the percentage of individuals in each drought damage category ranging from no visible sign of stress (1, green) to progressive signs of wilting or rolling and tissue necrosis to complete death of all aboveground biomass (9, black). Shown are examples of forbs (top) and grasses (bottom) with **a**, **c** high drought survival and less severe, late signs of drought stress and **b**, **d** with high mortality and early, strong drought damage in the four nutrient conditions (unfertilized control C, P addition, N addition and NP combined addition). For a plot including all species, see Fig. S4 and for damage categories, see Table S4

Under experimental drought, all species showed clear visual signs of drought damage, including wilting and tissue necrosis. However, the progression and the severity of drought damage varied substantially among species (Fig. 1, for all species, see Fig. S4). In some species, most individuals had little or no living aboveground biomass at the end of the 8-week drought period (e.g., *Ranunculus bulbosus *and* Poa trivialis*). In other species, individuals exhibited only extended drying of leaf tips, and most of their leaf area stayed alive throughout the drought (e.g., *Achillea millefolium *and* Helicotrichon pubescens*). Visual signs of drought damage were similar across nutrient treatments within species (Fig. 1, S4).

**Fig. 2:**
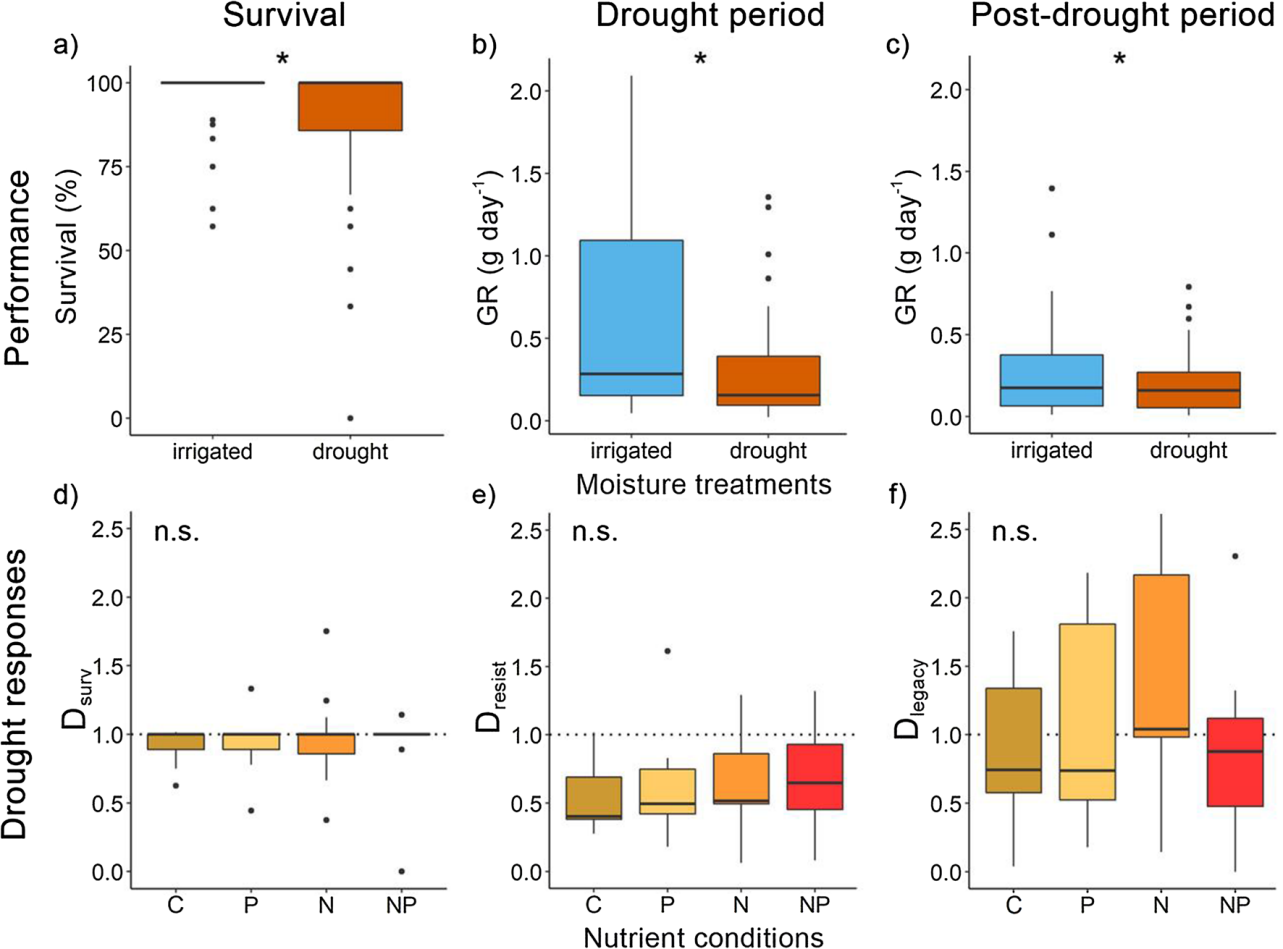
Performance in the drought and irrigation treatment, and corresponding drought responses under different nutrient conditions across 13 temperate grassland species. The top row depicts **a** survival, **b** growth during the drought period and **c**) growth in the post-drought period in the two moisture treatments, averaged across all species and nutrient treatments. The bottom row depicts **d** drought responses of survival (*D*_surv_), **e** drought resistance (*D*_resist_), and **f** drought legacy effects (*D*_legacy_) across all species in the four nutrient conditions (unfertilized control C, P addition, N addition, and combined NP addition). Drought responses < 1 (dotted line) indicate a lower performance in the drought relative to the irrigation treatment. Significant differences between the drought and irrigated treatment are highlighted by asterisks for *P* < 0.05; n.s. is not significant. Box plots show the medians (horizontal lines), 25th and 75th percentiles (boxes), and 1.5 × lower and upper quartiles (whiskers)

**Fig. 3:**
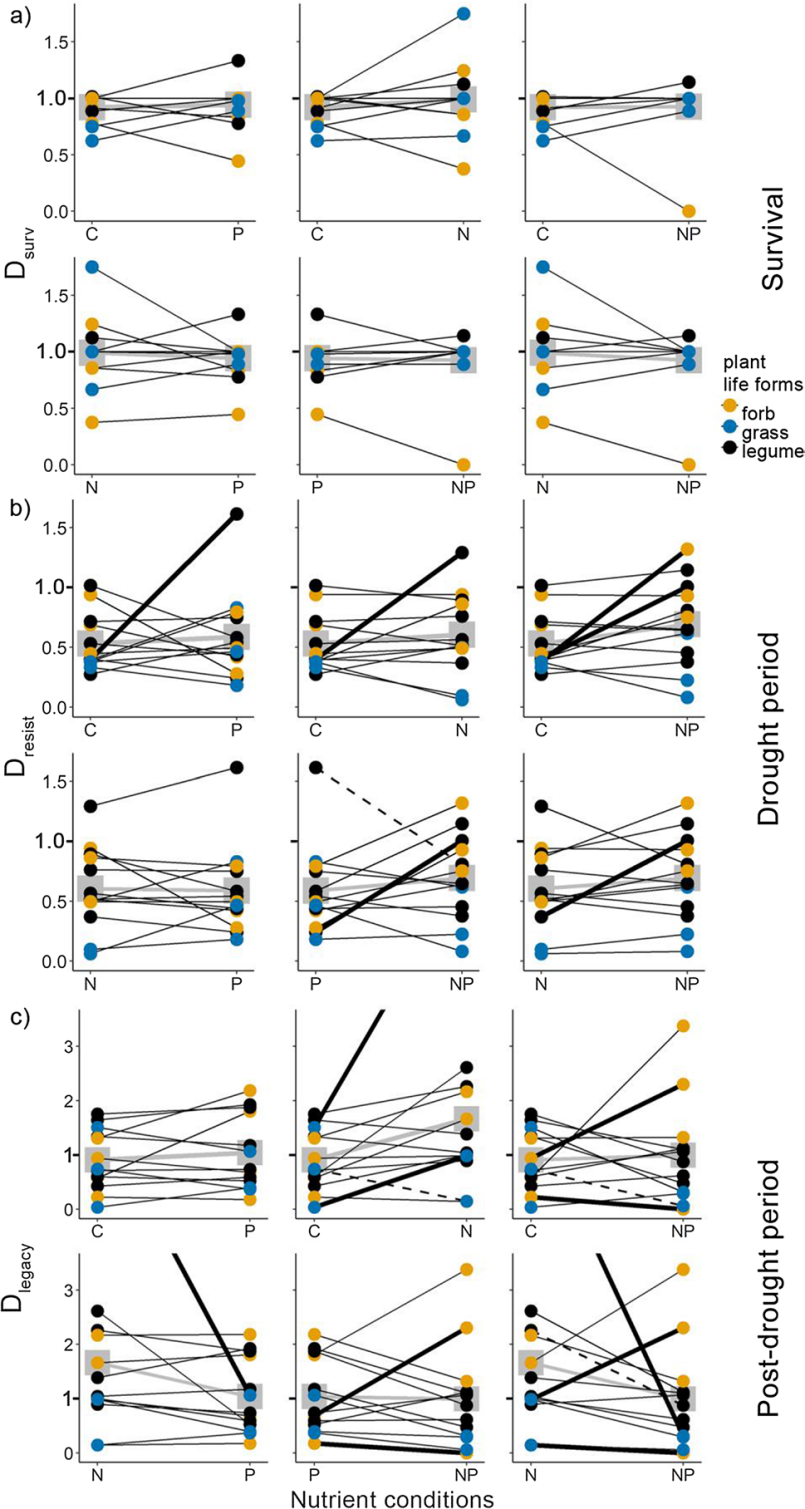
Changes of species **a** drought response of survival (*D*_surv_), **b** drought resistance of growth (*D*_resist_), and **c** drought legacy effects (*D*_legacy_) on growth in the different nutrient conditions. The dots represent the mean drought responses of the individual species under the respective nutrient conditions (unfertilized control C, P addition, N addition, and combined NP addition), colors indicate plant life forms (non-legume forbs in yellow, grasses in blue, and legumes in black). The lines connect the responses of each species under different nutrient conditions and show the change of drought response between treatments. Note the wide variation of slopes among species. Significant responses (*P* < 0.05) are highlighted with a solid, bold line and marginal responses (*P* < 0.1) are highlighted with dashed lines. Mean drought responses across all species are plotted in gray. Note that in a) *Ranunculus bulbosus* had no surviving individuals in the NP treatment (*D*_surv_ = 0) and in **c** the mean *D*_legacy_ of *Lotus corniculatus* in the N treatment (mean ± SD = 6.32 ± 1.94) is not shown, indicated by the cut line, for better visibility of the remaining species. Values < 1 indicate a lower performance in the drought relative to the irrigation treatment

Drought significantly reduced survival across species (*P* < 0.001; Table 2, Fig. 2a). Nevertheless, even under drought, survival was overall high (88% mean), with more than 60% of the species exhibiting drought survival above 90% (Fig. S5a). Furthermore, survival did not differ between nutrient conditions, and overall, no interaction of drought with nitrogen (N) or phosphorus (P) emerged (Table 2). Accordingly, survival responses to drought (*D*_surv_, survival in drought relative to irrigated conditions) did not differ among nutrient conditions or life forms (Table 3, Fig. 2d, Fig. S6a). However, drought survival responses varied between species from a substantial decrease (*D*_surv_ of 0.39 in *Ranunculus bulbosus)* to no response (*D*_surv_ of 1.08 in *Helicotrichon pubescens*). Only one species, *Ranunculus bulbosus,* showed the widely expected increase of drought vulnerability with nutrient availability, resulting in 100% mortality in NP-conditions (Fig. 3a).

**Table 2:** Effects of drought (D), nitrogen addition (N), and phosphorus addition (P), and their interactions on plant survival and on growth rates during the drought period and in the post-drought period

Fixed effects	*df*	Survival^a^			Drought period growth			Post-drought period growth		
		*χ*^*2*^	*P*		*χ*^*2*^	*P*		*χ*^*2*^	*P*	
D	1	**13.52**	** < 0.001**	**↓**	**46.74**	** < 0.001**	**↓**	**3.91**	**0.048**	**↓**
N	1	0.001	0.97		*3.07*	*0.08*	*↑*	**12.11**	**0.001**	**↑**
P	1	1.54	0.21		0.32	0.57		0.42	0.51	
DxN	1	0.002	0.96		0.48	0.49		0.15	0.70	
DxP	1	0.49	0.48		< 0.001	0.99		0.22	0.64	
NxP	1	0.90	0.34		0.19	0.66		0.03	0.86	
DxNxP	1	1.09	0.30		0.16	0.69		1.52	0.22	
Random effects										
(1| species)		*			*			*		
*R*^2^ marginal		0.13			0.11			0.06		
*R*^2^ conditional		0.19			0.78			0.65		
Transformation								sqrt		

Results are based on regression models (GLMM and LMM) and the post-drought GR was square-root-transformed to improve model assumptions

Given are degrees of freedom *df*, *χ*^2^ values, *P* values, marginal *R*^2^ (fixed effects), and conditional *R*^2^ (random and fixed effects)

Significant effects (*P* < 0.05) are indicated in bold, and marginal effects (*P* < 0.1) in cursive. The direction of significant and marginal effects is highlighted with arrows for increase (arrow pointing up) decrease (arrow pointing down). Significant random effects (*P* < 0.05) are highlighted with asterisks

^a^Survival was assessed over the drought and post-drought period combined, to account for possible resprouting

**Table 3:** Effects of N addition, P addition, and their interactions on drought response of survival (*D*_surv_), drought resistance (*D*_resist_), and drought legacy (*D*_legacy_)

Across species	*df*	Survival *D*_surv_		Drought period *D*_resist_		Post-drought period *D*_legacy_	
		*χ*^*2*^	*P*	*χ*^*2*^	*P*	*χ*^*2*^	*P*
N	1	0.30	0.59	1.58	0.21	1.36	0.24
P	1	0.20	0.65	1.00	0.32	1.47	0.23
NxP	1	0.83	0.36	0.05	0.83	*3.54*	*0.06*
Random effects (1| species)		*		*		*	
*R*^2^ marginal		0.01		0.03		0.07	
*R*^2^ conditional		0.50		0.41		0.47	
Transformation						log	

Species	*df*	Survival *D*_surv_		Drought period *D*_resist_		Post-drought period *D*_legacy_	
		*χ*^*2*^	*P*	*χ*^*2*^	*P*	*χ*^*2*^	*P*
Sp	12			**130.31**	** < 0.001**	**262.57**	** < 0.001**
N	1			4.29	0.04	0.001	0.982
P	1			1.26	0.26	**9.82**	**0.002**
SpxN	12			**27.01**	**0.01**	**46.69**	** < 0.001**
SpxP	12			*20.60*	*0.06*	**26.63**	**0.01**
NxP	1			0.73	0.39	**5.36**	**0.02**
SpxNxP	12			**45.43**	** < 0.001**	**34.26**	**0.001**
Random effects							
(1|Block)				*			
(1|Neighbor biomass)				*		*	
*R*^2^ marginal				0.36		0.20	
*R*^2^ conditional				0.51		log0.22	
Transformation				sqrt		sqrt	

Plant life forms	*df*	Survival *D*_surv_		Drought period *D*_resist_		Post-drought period *D*_legacy_	
		*χ*^*2*^	*P*	*χ*^*2*^	*P*	*χ*^*2*^	*P*
Lf	2	1.83	0.40	*5.71*	*0.06*	0.87	0.65
N	1	0.31	0.58	1.61	0.21	1.14	0.29
P	1	0.21	0.65	1.02	0.31	1.68	0.20
LfxN	2	3.53	0.17	*4.63*	*0.10*	0.26	0.88
LfxP	2	1.73	0.42	0.10	0.95	*5.20*	*0.07*
NxP	1	0.86	0.35	0.05	0.83	**4.13**	**0.04**
LfxNxP	2	2.20	0.33	1.97	0.37	2.41	0.30
Random effects							
(1| species)		*		*		*	
*R*^2^ marginal		0.15		0.26		0.16	
R^2^ conditional		0.58		0.48		0.56	
Transformation						sqrt	

Effects were analyzed across species, including species (Sp) or plant life form (Lf) as main effects, respectively, and their interactions. Significant effects (*P* < *0.05*) are indicated in bold; marginal effects *P* < 0.1 cursive. Given are degrees of freedom *df*, *χ*^2^ values, *P* values, marginal R^2^ (fixed effects), and conditional *R*^2^ (random and fixed effects)

Significant random effects (*P* < 0.05) are highlighted with asterisks and transformations are specified where applied. Species effects could not be tested as fixed effects in *D*_surv_, which was assessed at the species level

### Effects of nutrients on growth during drought

Across species, drought significantly reduced growth compared to the irrigated treatment (by 50%; *P* < 0.001, Fig. 2b). Nitrogen addition had a less pronounced positive effect on growth (+ 19%, *P* = 0.08) and phosphorus addition had no significant effect. No significant interaction of drought with N or P addition on growth emerged across species (Table 2). Consistently, across species N or P addition, or their interaction had no significant effect on drought resistance (*D*_resist_; Table 3, Fig. 2e).

Drought resistance (*D*_resist_) differed strongly across species (*P* < 0.001). Most importantly, however, nutrient conditions affected drought resistance differentially among species, with both the magnitude and the direction of the effects varying across species and between N and P addition (i.e., significant 3-way Sp × N × P interaction, *P* < 0.001, Table 3, Fig. 3b). For example, adding a single nutrient increased drought resistance in some species (up to 3.3-fold in *Poa trivialis*). In others, only combined NP addition increased drought resistance (about 2.5-fold in *Taraxacum sp.* and *Briza media* comparing unfertilized and NP). Yet, within most individual species, drought resistance did not differ significantly among nutrient conditions (Fig. 3b, Fig. S7a). Nutrients did not differentially affect drought resistance among life forms (i.e., no life form × nutrient interaction; Table 3, Figs. S6b, S7b).

### Legacy effects of drought on growth in the following growing season

Drought reduced growth not only during the drought but also in the following growing season (negative drought legacy effect). The growth rate in the spring after the drought (GR_post-drought_) was still affected by the previous moisture treatments, with a mean 27% reduction in plants that had experienced the experimental drought treatment compared to irrigated ones (*P* = 0.048, Table 2, Fig. 2c). Nitrogen addition still led to an overall growth enhancement (+ 30%, *P* = 0.001, Table 2), while phosphorus addition had no effect. Across species, nutrient conditions did not significantly affect legacy effects (*D*_legacy_, Table 3, Fig. 2f). However, as we had found for drought resistance, drought legacy effects again differed strongly among species and between N and P addition (i.e., significant 3-way interaction of Sp × N × P, *P* < 0.001, Table 3, Fig. 3c). Some species had reduced, but others even higher growth under the formerly droughted than irrigated conditions, depending on the nutrient condition (negative vs. positive drought legacy effect, i.e., *D*_legacy_ < / > 1, respectively). Overcompensation occurred in *Achillea millefolium* under combined NP addition (post-drought growth rate increased about 2.5-fold) and in *Lotus corniculatus* under nitrogen addition. Nitrogen addition also dampened negative drought legacy effects in *Trifolium repens* (but no overcompensation, Figs. 3c, S7c).

Among life forms, the effects of P addition on drought legacy effects differed (significant 2-way interaction Lf × P, *P* = 0.04, Table 3, Fig. S7d). Combined NP (but not P alone) led to negative legacy effects in legumes, while no nutrient effects were observed in grasses or non-leguminous forbs (Fig. S6c).

Species’ drought resistance and the drought legacy effects they exhibited were positively related (*R* > 0.64, *P* ≤ 0.02) under nitrogen addition (N, NP treatment). Species that suffered stronger during the drought event, i.e., had lower drought resistance, thus also exhibited more negative drought legacy effects, i.e., their growth was still more strongly reduced after drought. However, this pattern did not hold under unfertilized conditions and P addition. Instead, some highly drought resistant species exhibited negative legacy effects (e.g., *Poa trivialis with P addition*). In contrast, others had reduced growth under drought (i.e., low drought resistance) but recovered or even exhibited a positive drought legacy effect (e.g., *Helicotrichon pubescens *and* Achillea millefolium*).

The observed interspecific variation of the size and direction of nutrient effects on drought responses (*D*_surv_, *D*_resist_ or *D*_legacy_) was not associated with species’ habitat association to nutrients or moisture, their resource-use strategy, their biomass allocation to roots, or the strength of nutrient limitation (see Table S5).

### Species performance ranks under drought changed with nutrient addition

**Fig. 4:**
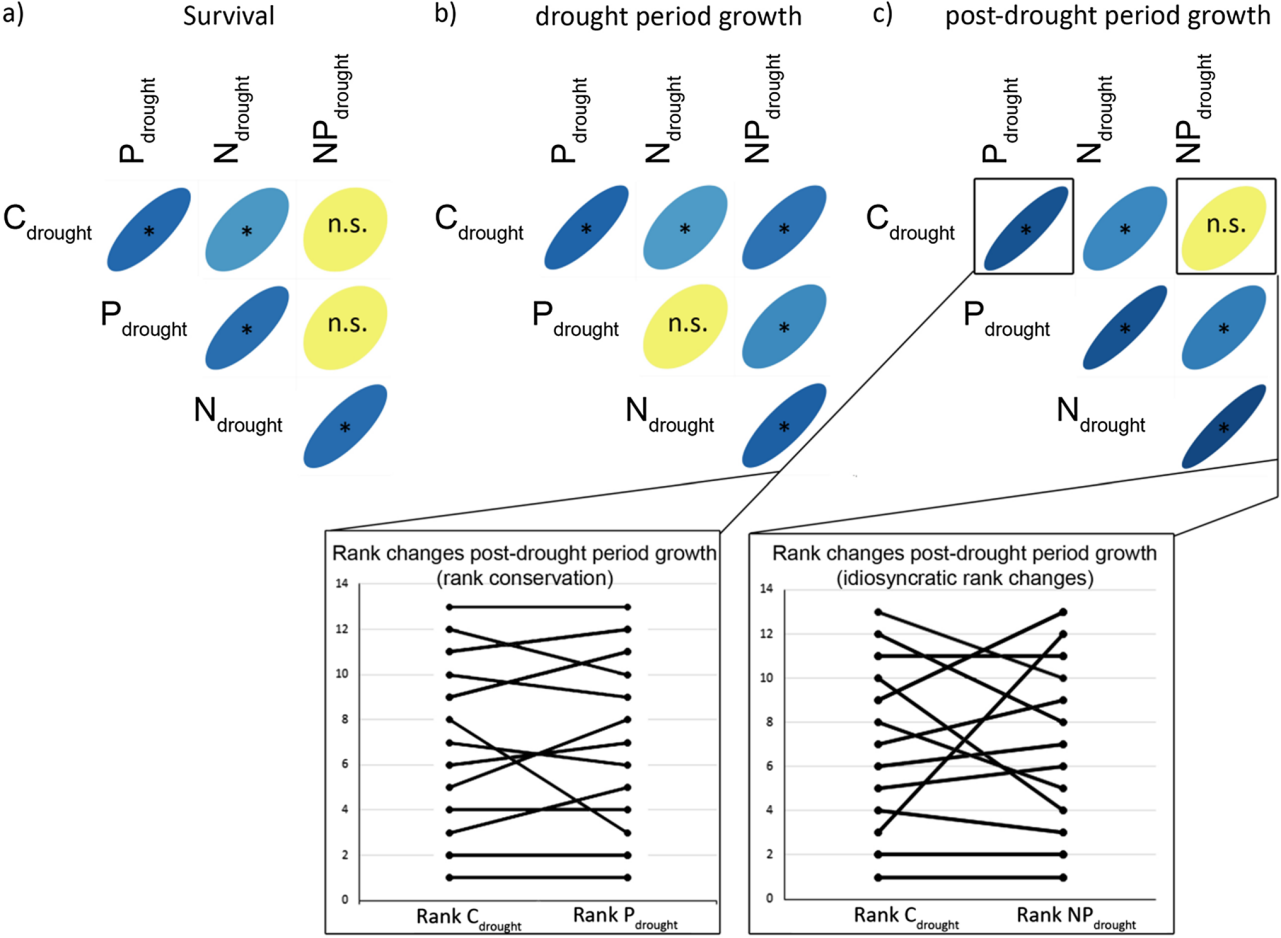
Species rank correlation coefficient of **a** survival, **b** growth during the drought period and **c** in the post-drought period between all pairwise combinations of the nutrient conditions within the drought treatment. The shape of the ellipse indicates the strength of the correlation, with slim ellipses indicating tight correlations. Significant (*P* < 0.05) correlation coefficients are highlighted with asterisks. Non-significant correlations (n.s., *P* > 0.05), indicating performance rank changes, are highlighted in yellow. In (**c**) rank changes of post-drought growth are shown for one example with rank conservation (i.e., positive significant correlation) and one example for idiosyncratic rank changes (n.s. correlation). Note that no negative correlations (rank reversals) emerged. For a version showing the correlations with the datapoints, see Fig. S8

Under drought conditions, species performance ranks changed idiosyncratically between several nutrient conditions (i.e., ranks were uncorrelated, *P* > 0.05, highlighted in yellow in Fig. 4). Species ranking of survival changed between combined NP addition compared to unfertilized control or P addition (Fig. 4a, NP *vs.* C, P), during the drought period ranking of growth changed with N addition (Fig. 4b, N vs. P) and after the drought period ranking of growth changed with combined NP addition (Fig. 4c, NP vs. C). Under irrigated conditions, species ranking of growth was conserved across all nutrient treatments (i.e., all positive correlations), both during the drought and in the post-drought period. No rank reversals (i.e., negative correlations) emerged.

## Discussion

Nutrient conditions affected drought responses differentially among common temperate grassland species, resulting in changed species performance ranks under drought. The experimental approach allowed for the first time to directly compare the combined effects of nutrients and drought on whole-plant performance across multiple temperate grassland species.

High nutrient availability, especially nitrogen, has been widely assumed to increase plant vulnerability to drought (e.g., Bobbink et al. 1998; Grime et al. 2000). However, our results and several previous studies (Carlsson et al. 2017; Hofer et al. 2017) do not support this notion. Neither drought resistance nor drought legacy exhibited an overall effect of nutrients, but effects varied strongly among species. Only one species (*Ranunculus bulbosus*) showed the widely assumed decrease of drought resistance and increased mortality with nutrient availability. In contrast, the remaining species with significant responses mainly exhibited an increase of drought resistance or a positive drought legacy effect with nutrient addition. Overall, our results indicate that there is no overall consistent effect of nutrients on drought responses of common temperate grassland species: Instead, nutrients enhance drought performance (i.e., dampen negative drought effects) in some species or render others more vulnerable to drought (i.e., amplify negative drought effects). However, in many of the investigated grassland species, the range of nutrients they experience in the habitat, even under fertilization, hardly affected drought responses even under intense drought conditions.

The wide variation of effects of nutrients on drought responses we observed was unrelated to species' life forms. Previous studies had found a positive effect of nitrogen addition on drought resistance of grasses and non-legume forbs, but not on legumes (Carlsson et al. 2017; Hofer et al. 2017) or nutrient addition intensifying negative drought effects in grasses, but not in forbs (Van Sundert et al. 2021). Instead, we observed strong variation even within plant life forms, including legumes. The variation of effects of nutrient on drought responses was also not related with species' habitat associations to nutrients or moisture availability, their resource-use strategy, investment into root tissue, or nutrient limitation. Thus, while we observed a wide variation of the effects of nutrients on drought responses across species and plant life forms, we are currently unable to explain this variation. Further, more detailed studies will be required to specifically address this question. Species idiosyncratic responses to drought under different nutrient conditions are instead likely mediated by differences across species in the phenotypic plasticity of traits related to drought responses. It is well known that trait expression of grassland species is strongly plastic in response to nutrients (e.g., Aerts and Chapin 2000). This has been shown for several traits relevant to plant drought resistance (e.g., biomass allocation, water use efficiency, and hydraulic conductivity; Godoy et al. 2011; Goldstein et al. 2013; Meyer-Grünefeldt et al. 2013; Chieppa et al. 2019) and should also extend to others. The direction and magnitude of trait changes may vary depending on the considered trait, across species and life forms, or on the nutrient added (Goldstein et al. 2013). Such plastic changes of trait expression and their coordination in response to nutrients lead to different and sometimes counterintuitive outcomes for whole-plant drought performance (Goldstein et al. 2013). Phenotypic trait changes in response to nutrients and drought can affect plant performance even after the drought and may underlie the drought legacy effects we observed (Reichmann and Sala 2014; De Boeck et al. 2018). However, the consequences of trait changes under nutrients for drought responses are hardly explored. An enhanced understanding of how traits and trait plasticity in response to nutrients influence drought responses in grassland species will contribute to understanding and predicting species, community, and ecosystem responses to combined effects of fertilization and drought. The comparative characterization of the differential effects of nutrients for whole-plant drought responses across species in this study will provide a baseline for evaluating the mechanisms underlying the observed idiosyncratic variation across species, and to unravel their importance for community and ecosystem responses.

Consistent with the variable species responses, the species performance hierarchy under drought changed under different nutrient conditions. Specifically, the ranking of species growth rates under drought changed with nitrogen addition, but also the ranking of survival and growth rates after drought were affected by combined NP addition. Such idiosyncratic changes in performance ranking may modify competitive hierarchies and thus lead to pervasive differences in how community composition and ecosystem function of temperate grasslands respond to drought in high versus low nitrogen habitats (Harpole and Tilman 2007; Peñuelas et al. 2013). The pronounced differences across common grassland species in how nutrients affect their drought responses, and the changes in performance hierarchies imply that the abundance of species with different responses should modulate how strongly and in which direction nutrients affect community and ecosystem responses to drought. Thus, the composition of plant communities should be a decisive factor for scaling responses from the species to the ecosystem level (Grant et al. 2014). Differential drought responses of individual species to nutrients, as we found in this study, may therefore also underlie the contrasting effects previously shown at the community level, ranging from amplifying to dampening. Resolving the responses of individual species to combined stressors thus remains one prerequisite to improve our understanding of community and ecosystem-level responses to crucial global change drivers (Kardol et al. 2012). Nevertheless, community and ecosystem responses to drought and fertilization are not only shaped by individual species responses but also by species interactions (e.g., competition, herbivory or mutualistic interactions) as well as further abiotic factors or land use (e.g., light, mowing, e.g., Suttle et al. 2007). These additional factors make prediction and the development of appropriate mitigation and management strategies even more difficult.

## Conclusion

Climate change and land use simultaneously impose multiple stressors on plants through increased drought and fertilization. Differential effects of nutrients nutrient on drought resistance and drought legacy effects across individual species, as we observed in our study, may lead to pervasive consequences for grassland composition and productivity along nutrient and land-use gradients. Idiosyncratic species responses complicate predictions of community and ecosystem responses to climate and land-use changes. Unraveling the mechanisms that render species vulnerable to drought under different nutrient conditions, determining how responses are altered by species interactions, and examining how they translate into community and ecosystem-level responses remain important topics for future research.

## Supplementary Information

Below is the link to the electronic supplementary material.

Supplementary file1 (DOCX 4941 KB)

## Data Availability

Data were deposited in the Dryad Digital Repository https://doi.org/10.5061/dryad.fqz612jws.
