# Supplementary material for: Nutrient effects on drought responses vary across common temperate grassland species
Source: Oecologia. 2023 May 5;202(1):1–14. doi: 10.1007/s00442-023-05370-5 (PMC10229692; doi:10.1007/s00442-023-05370-5)
Supplement: Supplementary file 1 — Supplementary file1 (DOCX 4941 KB) [file 442_2023_5370_MOESM1_ESM.docx]

**Supplementary Information**

**Nutrient effects on drought responses vary across common temperate grassland species**

Carola Kiene^1*^, Eun-Young Jung^1^, Bettina M.J. Engelbrecht^1, 2^

Journal: Oecologia

Affiliations:

^1^Functional and Tropical Plant Ecology, Bayreuth Centre of Ecology and Environmental Research (BayCEER), University of Bayreuth, 95440Bayreuth, Germany

^2^Smithsonian Tropical Research Institute, Apartado 0843-03092, Balboa, Ancon, Republic of Panama

*corresponding author

Email address of corresponding author:

Carola.Kiene@uni-bayreuth.de

## **Supplemental Figures**


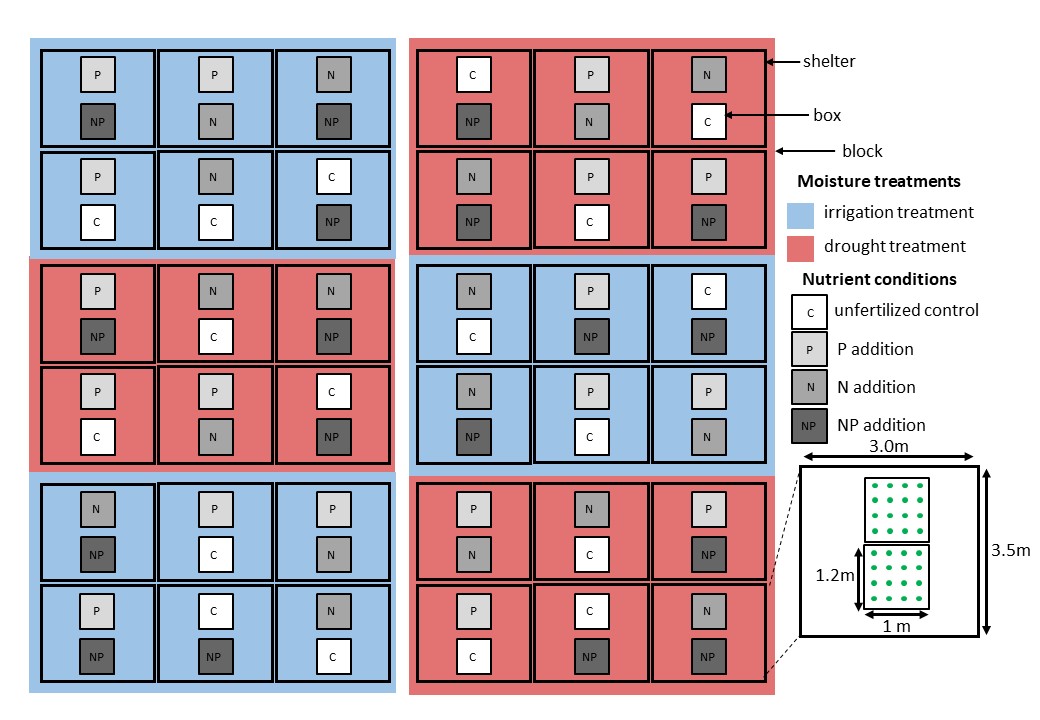


**Fig. S1** Schematic overview of the experimental design. Four nutrient conditions (unfertilized control C, phosphorous addition P, nitrogen addition N, combined NP addition) crossed with two moisture treatments (drought, irrigation) were applied to 13 species in 72 large boxes. Boxes were located in six blocks, with three blocks per moisture treatment. Three boxes per nutrient treatment were randomly located within each block. During the moisture treatments, all boxes were covered by rainout shelters, with two boxes below each shelter. One individual of each species was plantedper box, randomly assigned to positionsin a 20 cm x 20 cm grid


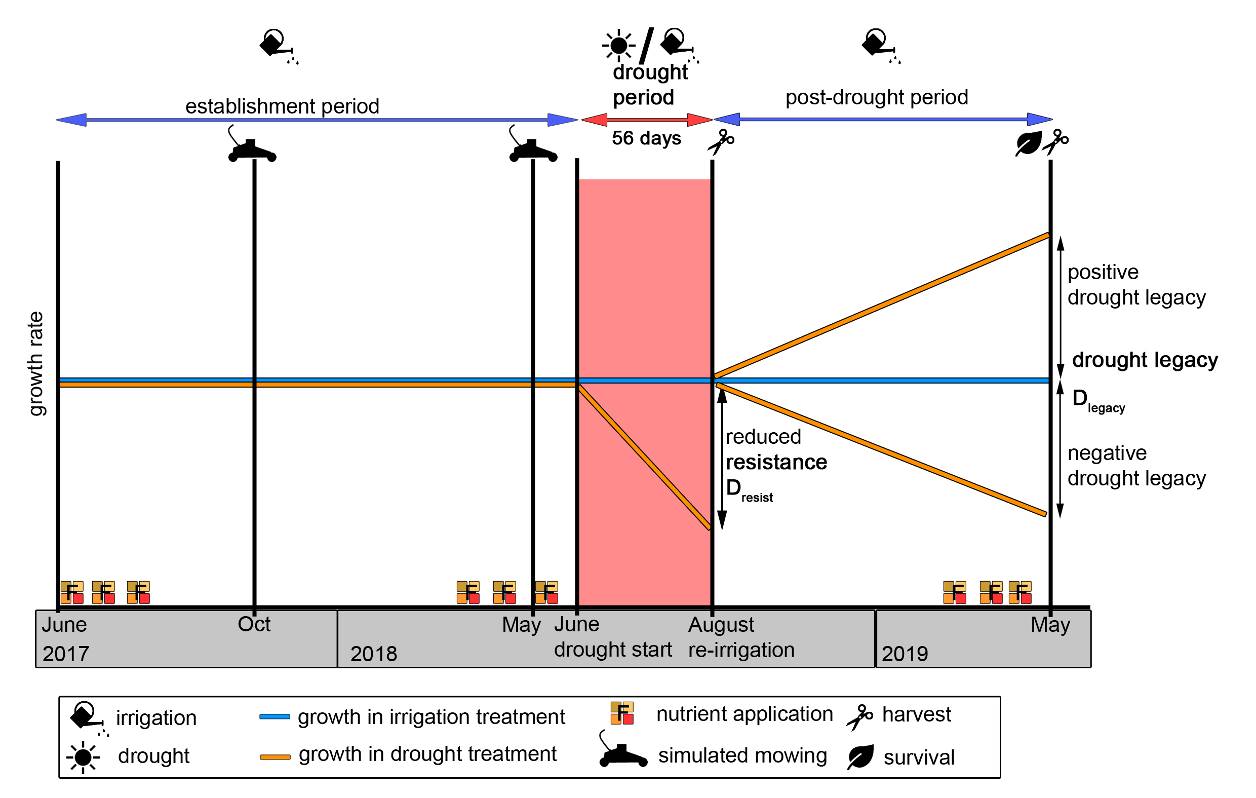


**Fig. S2** Schematic overview of the timeline of the experimental periods and the assessment of performance parameters. After a 12-month establishment period under the four different nutrient conditions (all plants fully irrigated) the two moisture treatments (drought, irrigation) were applied for 8 weeks (drought period, highlighted in red). During the drought period, all plants were covered by rain-out shelters. There after, rain-out shelters were removed and all plants were kept under full irrigation until the next growing season (post-drought period). Plant growth (GR) was assessed as aboveground biomass increase over the drought period (for drought resistance) and over the post-drought period (for drought legacy) based on harvests of the individual plants. For simplicity, the scheme shows the growth rate in the two moisture treatments (irrigation (blue) and drought (orange)) for one species in one nutrient treatment, and assumes constant growth under irrigated conditions. Whole-plant survival was assessed after the post-drought period to account for potential resprouting of plants. Performance responses to drought were assessed asperformance under drought relative to irrigated conditions for growth during the drought period (D_resist_), growth in the post-drought period (D_legacy_) and drought survival (D_surv_)

**
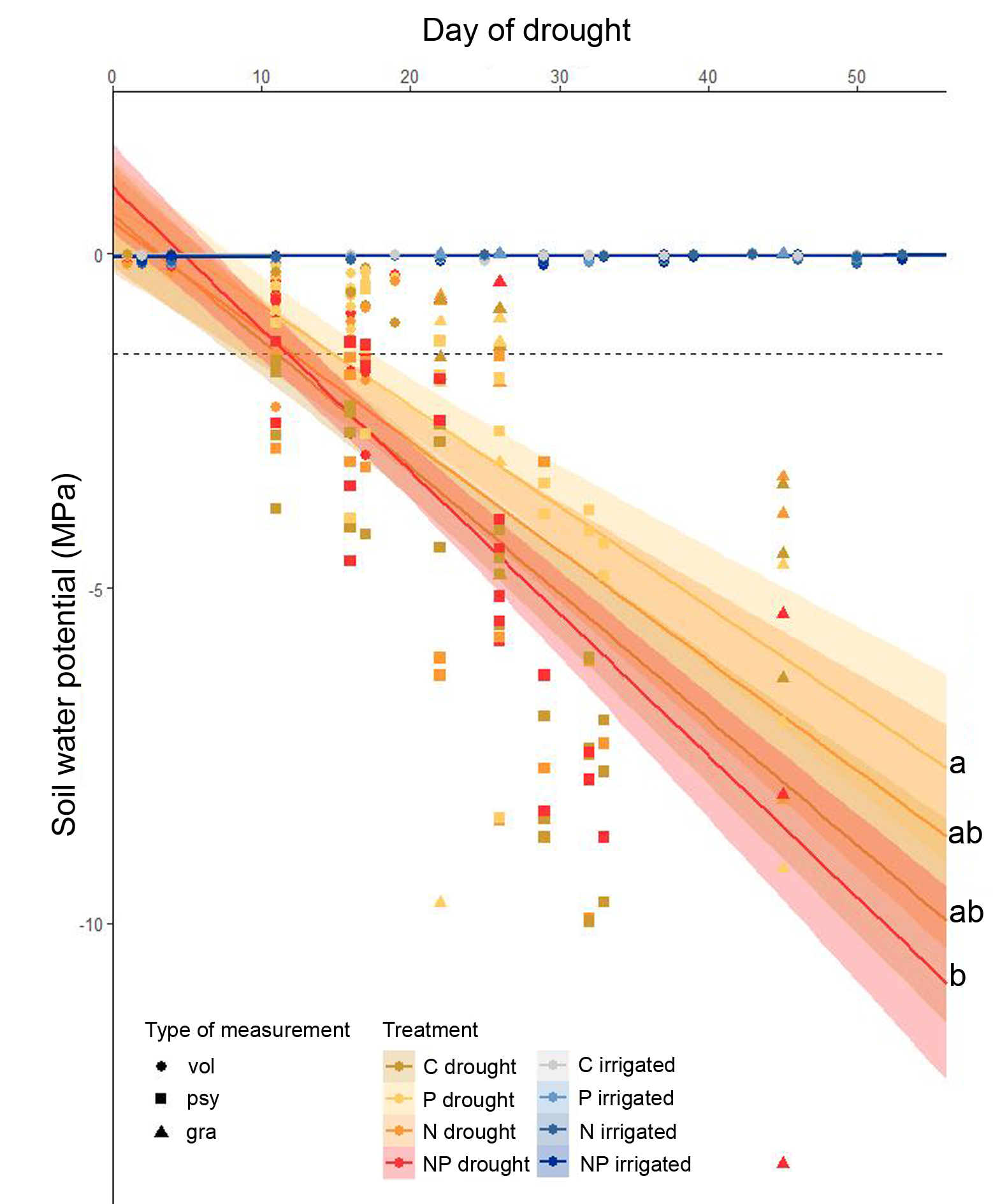
**

**Fig. S3** Soil water potentials in the drought treatment (yellow/red) and irrigation treatment (blue) over the 8-week moisture treatments. Regression lines indicate the decrease of soil water potential under drought in the different nutrient conditions (colors see legend, means ± confidence intervals). Different letters indicate significant differences (*P<0.05*) among nutrient conditions, i.e., the soil in the P-addition dried out slower than in the NP-addition. The dashed horizontal line indicates the permanent wilting point at -1.5MPa. Soil water potentials in the upper 20cm were assessed with a compilation of methods, indicated by the symbols (vol: volumetric, psy: psychrometric, gra: gravimetric, for details, see Methods S1). Volumetric and gravimetric water contents were converted to water potentials based on a water retention curve of the soil in the experiment (WP4C Dewpoint Potentia Meter, METER Group, Munich, Germany)

**a)

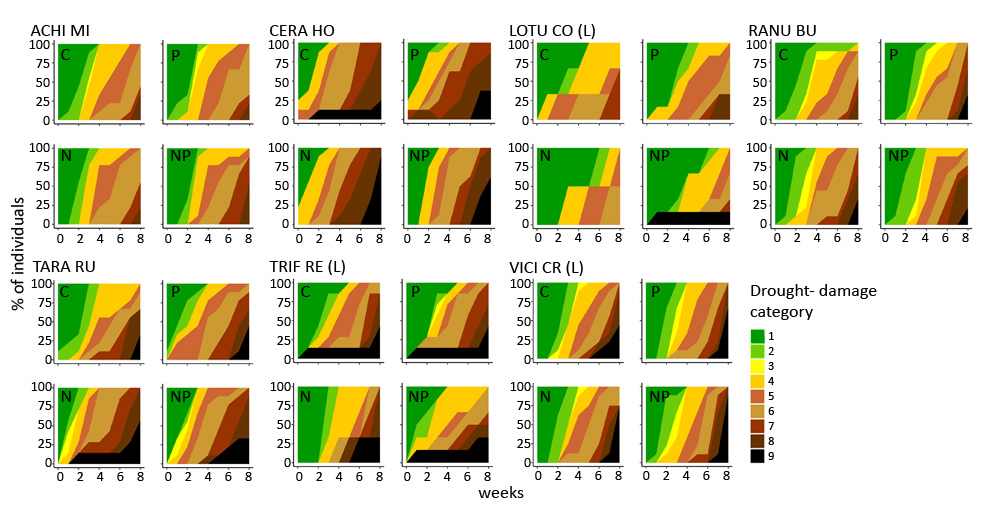
Fig. S4b)

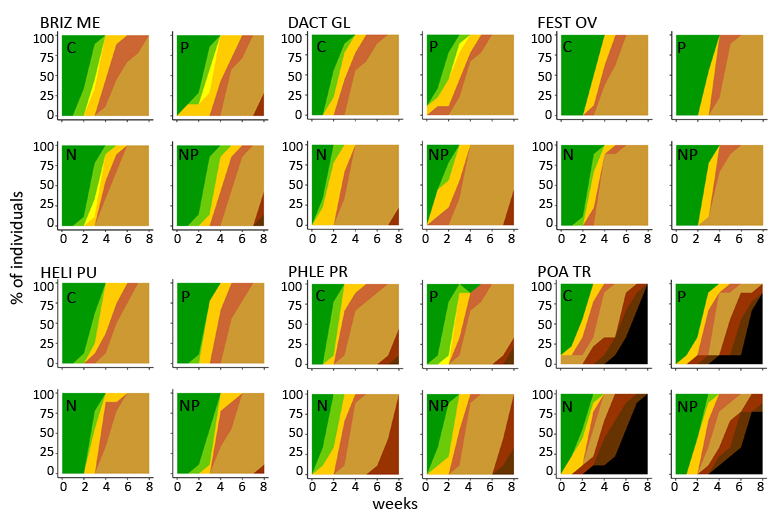
**

**Fig. S4 continued**

**Fig. S4** Progression of visual drought damage in the four different nutrient conditions (unfertilized control C, phosphorous addition P, nitrogen addition N, combined NP addition), over the 8-week experimental drought period. Shown is the percentage of individuals in each drought damage category (Table S4) ranging from no visual sign of stress (1, green) to progressive signs of wilting or rolling, and tissue necrosis to complete death of all aboveground biomass (9, black) for the 13 temperate grassland study species. Species are sorted alphabetically, separately for a) forbs and legumes (indicated with (L) after the species code) and b) grasses. For species codes, see Table 1

**Figure S5**

**
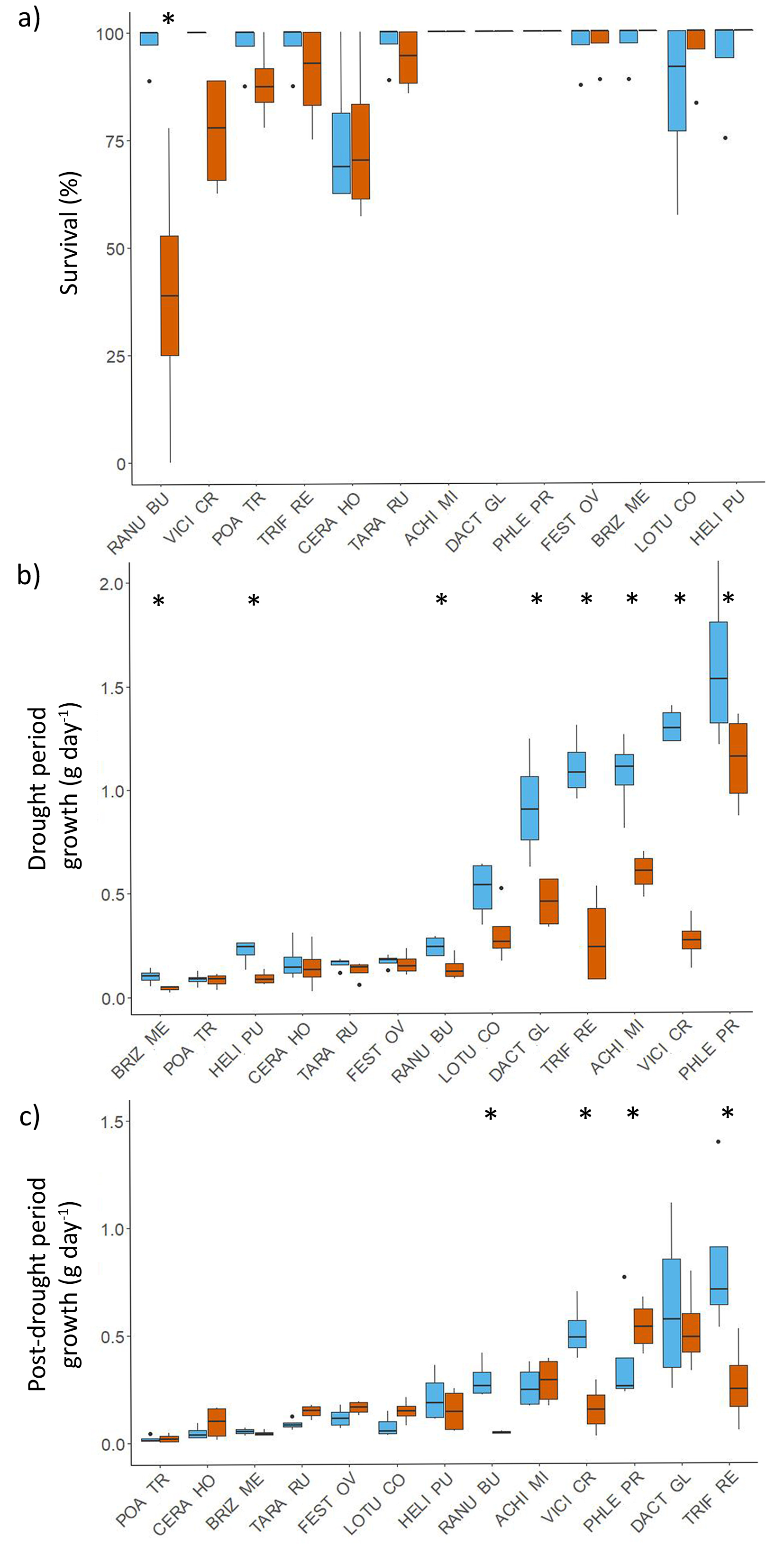
**

**Figure S5:** Performance of each of the 13 temperate grassland species in the drought treatment (orange) and the irrigation treatment (blue). Given are a) survival, b) growth during the drought period, and c) growth during the post-drought period averaged across nutrient conditions. Significant differences (*P<0.05*) between the drought and irrigation treatment within species are highlighted by asterisks. The black solid line indicates the median value, the edges of the boxes reach the first and third quartiles, and the whiskers extend to 1.5 times the interquartile range. Species are ordered from low to high survival and growth, for species’ codes see Table 1

**
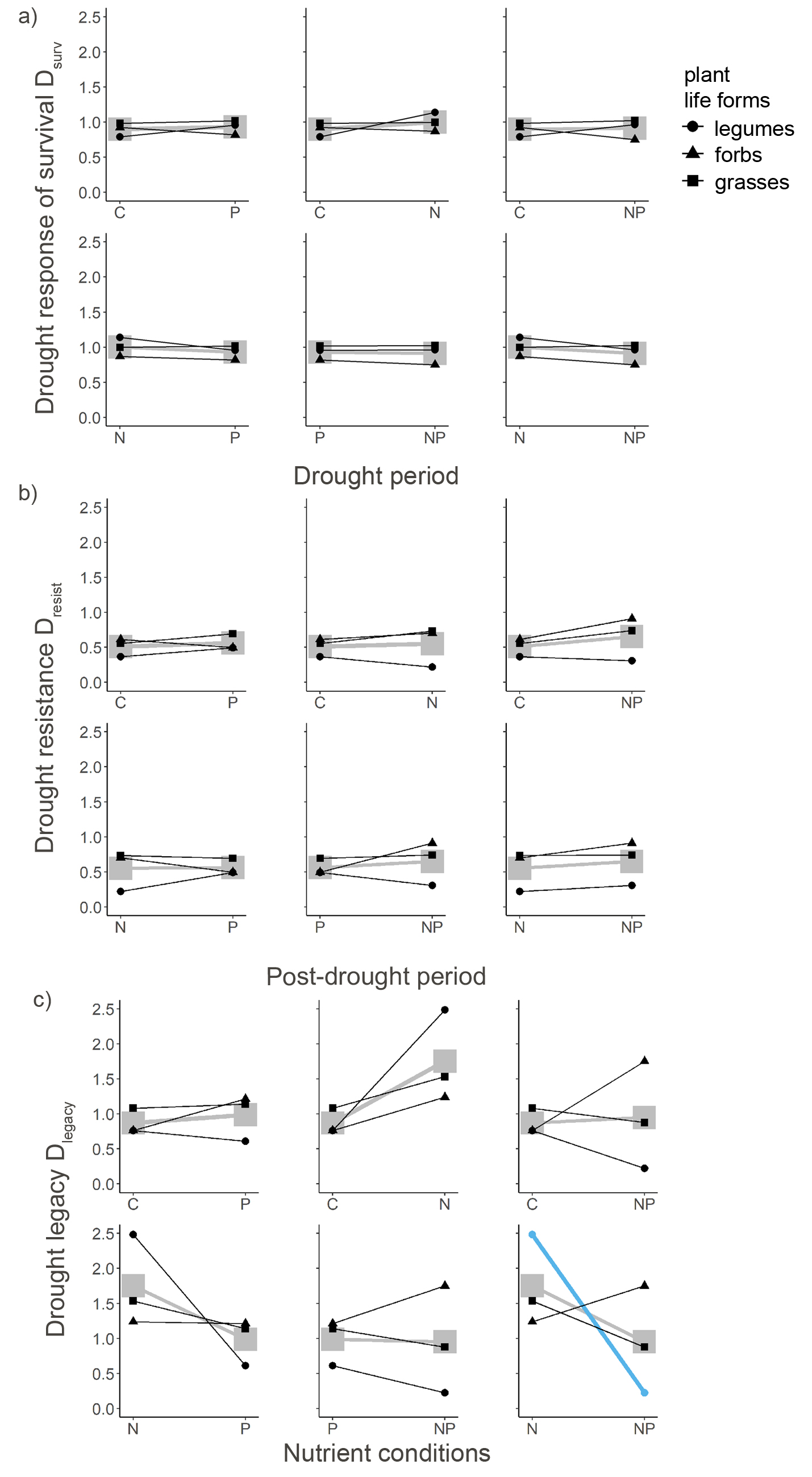
**

**Figure S6:** Drought responses of the different plant life forms (legumes, non-legume forbs and grasses) under different nutrient conditions (unfertilized control C, phosphorous addition P, nitrogen addition N, combined NP addition). Shown are a) survival response (D_surv_), b) drought resistance (D_resist_) and c) drought legacy effects (D_legacy_). Black symbols represent mean drought responses of the plant life forms under the different nutrient conditions. Values < 1 indicate a lower performance in the drought relative to the irrigation treatment. Lines connect responses of each plant life form between nutrient conditions, i.e., the slopes indicate the change of drought responses across nutrient conditions. Significant changes are highlighted in blue (*P < 0.05*). Mean drought responses across plant life forms are plotted in grey in the background


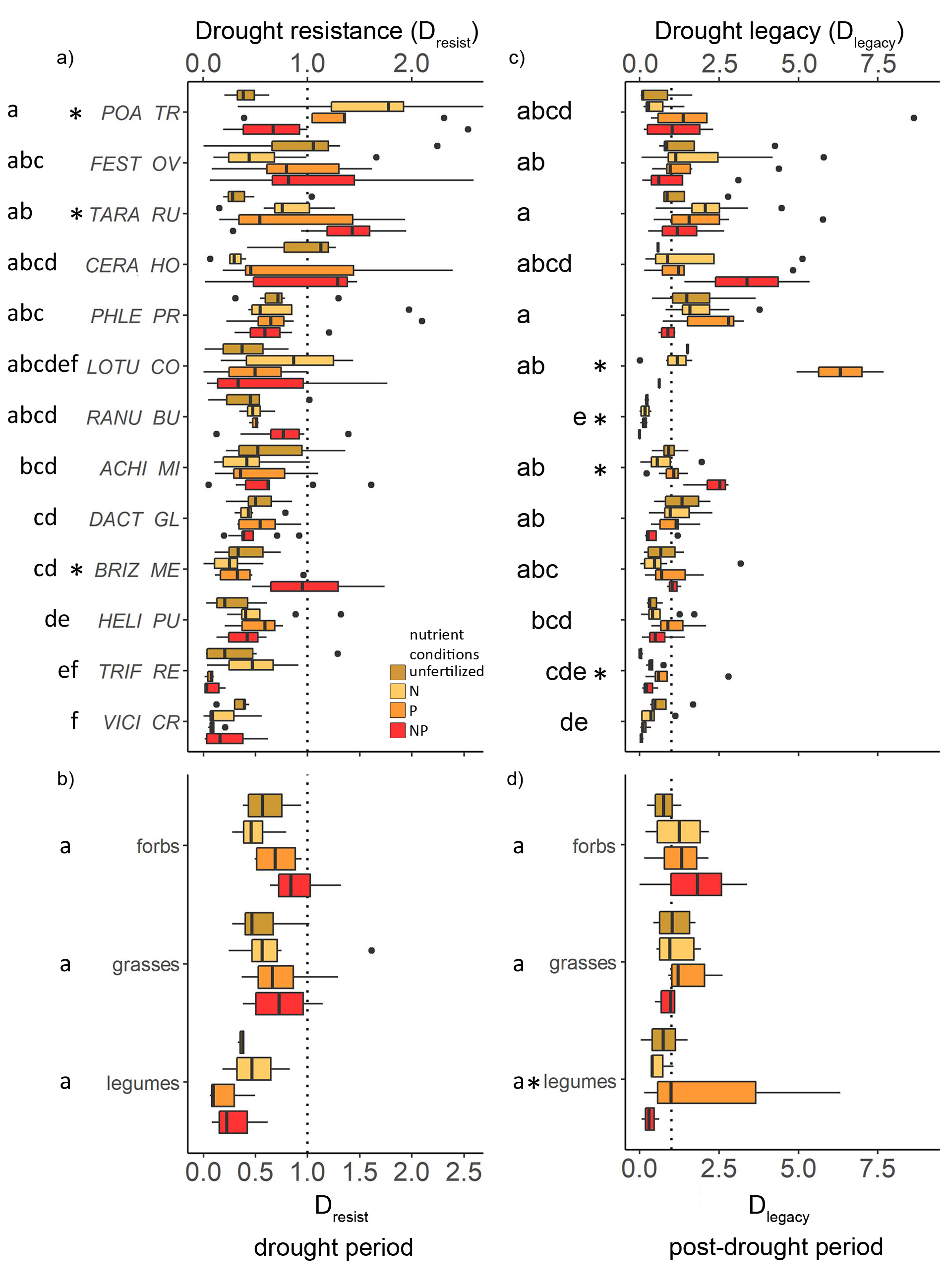


**Fig. S7** Drought resistance a), b) and drought legacy effects c), d) on growth under the four different nutrient conditions for the 13 temperate grassland species. Results are given at the level of individual species a), c) and life forms b), d). Values < 1 (dashed line) indicate a reduction in growth in the drought relative to the irrigation treatment. Species are ordered according to their mean drought resistance (D_resist_). Significant differences (*P < 0.05*) among nutrient conditions within species or life forms are indicated by asterisks. Species and life forms denoted with different letters are statistically different (*P < 0.05*) in their drought response according to pairwise comparison test using Holm's sequential Bonferroni procedure for multiple comparisons (78 and 3 comparisons, respectively). For species codes, see Table 1


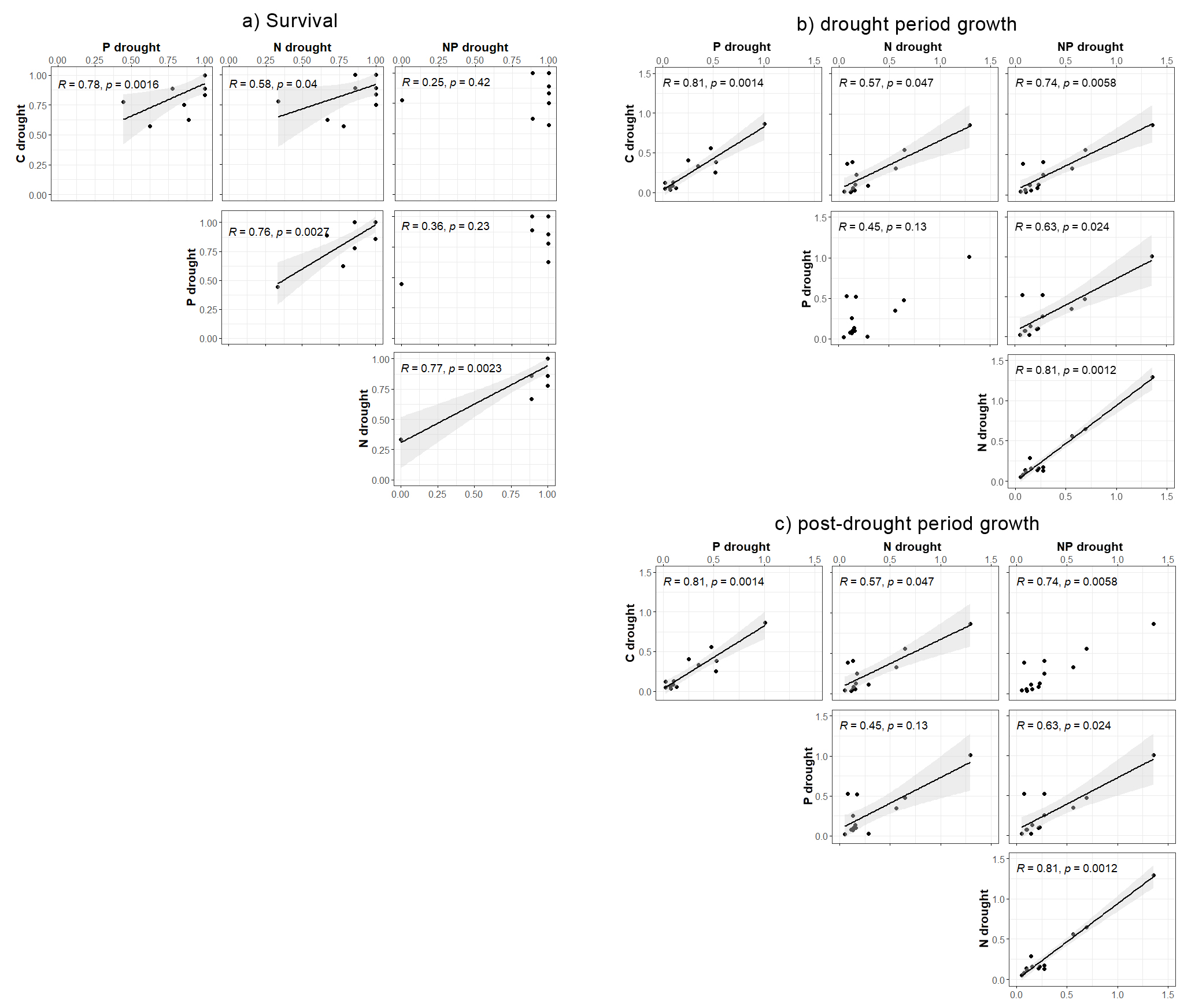
**Fig. S8**

**Fig. S8** Species rank correlations of a) survival, b) growth during the drought period and c) in the post-drought period between all pairwise combinations of the nutrient conditions within the drought treatment. Correlation coefficients and P-values are given for all correlations, lines and confidence intervals are shown for significant spearman correlations (P < 0.05). This is a modified version of Fig. 4, showing the datapoints. Non-significant correlations (n.s., *P > 0.05*), indicating performance rank changes

## **Glossary**

**Glossary S1** Glossary of terms and variable names, used abbreviations and their description

| **Terms and variable names** | **Abbrev** | **Definition** |
| --- | --- | --- |
| ***Experimental periods*** |  |  |
| establishment period |  | ~1 year period of plant establishment under the different nutrient treatments before implementation of the moisture treatments (June 2017-June 2018); all boxes were irrigated |
| drought period |  | period during which the moisture treatments (drought and irrigation) were applied (56 days, June-August 2018) |
| post-drought period |  | period after the moisture treatments were terminated and plants were allowed to recover until the following growing season (August 2018-May 2019); all boxes were irrigated |
| ***Nutrient treatments*** | | four nutrient treatments were applied yearly |
| phosphorus addition | P | equivalent of 50kg P ha^-1^yr^-1^ |
| nitrogen addition | N | equivalent of 100kg N ha^-1^yr^-1^ |
| NP addition | NP | combined addition of nitrogen and phosphorus |
| unfertilized control | C | no addition of N or P |
| ***Moisture treatments*** | | two soil moisture treatments were applied in the drought period |
| drought treatment |  | In half of the boxes, irrigation was discontinued under rainout shelters until most plants showed severe necrosis (56 days). |
| irrigation treatment |  | irrigation was continued throughout the drought period, adjusting irrigation levels to avoid visual signs of drought stress |
|  |  |  |
| ***Performance parameters*** | |  |
| growth | GR | absolute growth rate, assessed at the individual level as increase of aboveground biomass per time between two harvests. Growth rates refer to surviving individuals^1^ |
| growth during drought period | GR_drought_, GR_irrigated_ | growth rate during the drought period, i.e., between the pre- and post-drought harvest (May 2018 – August / September 2018) for both drought and irrigation treatments (GR_drought_ and GR_irrigated_, respectively), used to assess drought resistance |
| growth during post-drought period | GR_post-drought_, GR_post- irrigated_ | growth rate in the post-drought recovery period, i.e., between the post-drought and the final harvest (August / September 2018 - May 2019, for both formerly drought and irrigation treatments, used to assess drought legacy effects^1^ |
| drought survival | S_drought_, S_irrigated_ | whole-plant drought survival assessed after the post-drought period to account potential resprouting from surviving of belowground meristems, used to assess drought survival response |
| ***Drought responses*** | |  |
| drought resistance | D_resist_ | ratio of growth under the drought relative to irrigation treatment during the drought period |
| drought legacy | D_legacy_ | ratio of growth in the post-drought period of formerly droughted plants to formerly irrigated plants (i.e., that had not experienced drought stress throughout the experiment) ^1^ |
| drought survival response | D_surv_ | ratio of survival for each species in the drought relative to the irrigation treatment |

^1^ based on surviving individuals except for *Ranunculus bulbosus* in the NP, drought treatment, where no individuals survived. Here, all individuals were considered for GR_NP-drought_. In this case GR_post-drought_ and D_legacy_ of NP treatment were zero, due to no surviving individuals.

## **Supplemental Tables**

**Table S1** Monthly rainfall was considerably lower and monthly average temperature higher during the experimental drought period (June-August 2018) compared to the respective long-term averages (1981-2010). Data are from the Ecological Botanical Garden (EBG), University of Bayreuth

|  | **Rainfall (mm)** | | **Temperature (°C)** | |
| --- | --- | --- | --- | --- |
|  | 2018 | Long-term average | 2018 | Long-term average |
| June | 29 | 72 | 17.8 | 15.4 |
| July | 37 | 92 | 19.7 | 17.6 |
| August | 15 | 64 | 20.3 | 16.6 |
| Mean ±sd | 27±11.1 | 76±14.4 | 19.3±1.3 | 16.5±1.1 |

**Table S2** Effects of nitrogen addition (N), phosphorus addition (P), and their interactions on leaf and soil nutrient contents a) before the drought and b) after the drought period. After the drought period, drought effects (D) are additionally included. Given are degrees of freedom (df), χ^2^ values, *P-*values, marginal R^2^ (fixed effects) and conditional R^2^ (random and fixed effects). The direction of significant and marginal effects is highlighted with arrows for increase (arrow pointing up) decrease (arrow pointing down). Significant effects (P *< 0.05*) are highlighted in bold and marginal effects (*P <0.1*) in cursive. Species was included as random effect in analyses of leaf nutrient contents and was consistently significant. No random effects were included in the analyses of soil nutrient contents. Leaf nutrient contents were analyzed for 3 individuals in each species in each nutrient – moisture treatment combination, and soil nutrient contents were analyzed for 6 boxes per nutrient – moisture treatment combination

| a**) before the drought period** | | | | | | | | | | | **b) after the drought period** | | | | | | | | | | |  |
| --- | --- | --- | --- | --- | --- | --- | --- | --- | --- | --- | --- | --- | --- | --- | --- | --- | --- | --- | --- | --- | --- | --- |
| **Leaf nutrients** | | **N** | | | | | | **P** | | | **Leaf nutrients** | | **N** | | | | | | **P** | | |  |
| **Fixed effects** | **df** | *χ^2^* | | *P* | |  | | *χ^2^* | *P* |  | **Fixed effects** | **df** | *χ^2^* | | *P* | |  | | *χ^2^* | *P* |  |  |
| **N** | 1 | **52.46** | | **<0.001** | | **↑** | | **12.33** | **<0.001** | **↓** | **N** | 1 | 1.41 | | 0.23 | |  | | **33.00** | **<0.001** | **↓** |  |
| **P** | 1 | 0.4 | | 0.53 | |  | | **10.04** | **0.002** | **↑** | **P** | 1 | 0.25 | | 0.62 | |  | | **9.14** | **0.002** | **↑** |  |
|  |  |  | |  | |  | |  |  |  | **D** | 1 | 2.01 | | 0.16 | |  | | **5.39** | **0.02** | **↓** |  |
| **NxP** | 1 | 0.001 | | 0.97 | |  | | 0.06 | 0.81 |  | **NxP** | 1 | 0.03 | | 0.86 | |  | | 0.62 | 0.43 |  |  |
|  |  |  | |  | |  | |  |  |  | **DxN** | 1 | *3.06* | | *0.08* | |  | | 1.96 | 0.16 |  |  |
|  |  |  | |  | |  | |  |  |  | **DxP** | 1 | 1.64 | | 0.2 | |  | | 0.56 | 0.46 |  |  |
|  |  |  | |  | |  | |  |  |  | **DxNxP** | 1 | 1.32 | | 0.25 | |  | | 1.61 | 0.2 |  |  |
| **R^2^conditional** |  | 0.90 | | |  | | | 0.73 | | | **R^2^conditional** |  | 0.82 | | | | | | 0.84 | | |  |
| **R^2^ marginal** |  | 0.05 | | |  | | | 0.06 | | | **R^2^ marginal** |  | 0.02 | | | | | | 0.08 | | |  |
| **Soil nutrients** | | **NO_3_^-^** | | | **NH_4_^+^** | | | **P** | | | **Soil nutrients** | | **NO_3_^-^** | | | **NH_4_^+^** | | | **P** | | |  |
|  |  |  |  |  |  |  |  |  |  |  |  |  |  |  |  |  |  |  |  |  |  |  |
| **Fixed effects** | **df** | *χ^2^* | *P* |  | *χ^2^* | *P* |  | *χ^2^* | *P* |  | **Fixed effects** | **df** | *χ^2^* | *P* |  | *χ^2^* | *P* |  | *χ^2^* | *P* |  |  |
| **N** | 1 | 1.60 | 0.21 |  | 0.53 | 0.47 |  | 0.78 | 0.38 |  | **N** | 1 | 0.79 | 0.37 |  | **4.94** | **0.03** | **↑** | 0.58 | 0.45 |  |  |
| **P** | 1 | 0.91 | 0.34 |  | 0.15 | 0.70 |  | *3.09* | *0.08* | ↑ | **P** | 1 | 0.70 | 0.40 |  | 0.05 | 0.83 |  | 1.37 | 0.24 |  |  |
|  |  |  |  |  |  |  |  |  |  |  | **D** | 1 | **4.89** | **0.03** | **↑** | 1.36 | 0.24 |  | *3.44* | *0.06* | ↑ |  |
| **NxP** | 1 | 1.40 | 0.24 |  | 1.59 | 0.21 |  | 2.65 | 0.10 |  | **NxP** | 1 | 0.66 | 0.41 |  | 0.11 | 0.74 |  | 0.01 | 0.94 |  |  |
|  |  |  |  |  |  |  |  |  |  |  | **DxN** | 1 | 1.54 | 0.21 |  | 0.13 | 0.72 |  | <0.001 | 0.99 |  |  |
|  |  |  |  |  |  |  |  |  |  |  | **DxP** | 1 | 0.05 | 0.82 |  | 0.10 | 0.75 |  | 0.13 | 0.72 |  |  |
|  |  |  |  |  |  |  |  |  |  |  | **DxNxP** | 1 | 0.14 | 0.71 |  | 1.76 | 0.18 |  | 0.84 | 0.36 |  |  |
| **R^2^ marginal** |  | 0.18 | | | 0.11 | | | 0.23 | | | **R^2^ marginal** |  | 0.35 | | | 0.35 | | | 0.28 | | |  |

**Table S3** Leaf and soil nutrient concentrations a) before and b) after the drought period in the four nutrient conditions (unfertilized control (C), nitrogen addition (N), phosphorus addition (P) and combined NP addition). Nutrient concentrations (mean ± sd, per dry weight) are given for leaf nutrients in mg g^-1^ and for soil nutrients in mg  kg^-1^

| **a) before the drought period** | | | | **b) after the drought period** | | | | | |
| --- | --- | --- | --- | --- | --- | --- | --- | --- | --- |
|  | | | | **irrigation treatment** | | | **drought treatment** | | |
| **Leaf nutrients**  **(mg g^-1^)** | **N** | | **P** | **N** | | **P** | **N** | | **P** |
| **C** | 22.57±9.33 | | 3.7±1.1 | 18.68±7.18 | | 3.92±1.26 | 15.23±6.53 | | 3.77±1.68 |
| **P** | 22.91±9.26 | | 4.0±1.5 | 17.38±8.51 | | 4.10±1.45 | 16.93±5.36 | | 4.06±1.56 |
| **N** | 26.62±8.96 | | 3.2±0.9 | 16.05±7.44 | | 3.25±1.06 | 16.17±5.41 | | 3.07±0.97 |
| **NP** | 26.99±7.56 | | 3.6±1.0 | 16.38±9.72 | | 3.86±1.02 | 16.67±7.34 | | 3.26±1.18 |
| **Soil nutrients**  **(mg kg^-1^)** | **NO_3_^-^** | **NH_4_^+^** | **P** | **NO_3_^-^** | **NH_4_^+^** | **P** | **NO_3_^-^** | **NH_4_^+^** | **P** |
| **C** | 24.5±6.78 | 0.82±0.29 | 17.56±4.08 | 16.77±3.90 | 1.26±0.11 | 13.67±3.05 | 17.73±2.20 | 0.96±0.18 | 17.87±5.20 |
| **P** | 24.83±5.10 | 0.72±0.11 | 17.96±6.1 | 15.23±4.28 | 1.13±0.3 | 16.57±1.97 | 19.37±3.86 | 1.23±0.28 | 18.80±1.71 |
| **N** | 23.12±4.08 | 0.74±0.14 | 14.36±2.94 | 12.73±1.67 | 1.46±0.46 | 13.78±4.52 | 22.17±8.95 | 1.40±0.41 | 14.83±5.31 |
| **NP** | 33.07±15.76 | 0.94±0.43 | 24.62±12.35 | 17.43±3.49 | 1.57±0.38 | 13.73±1.46 | 26.13±13.52 | 1.26±0.12 | 19.23±7.28 |

**Table S4** Drought-damage categories used in the visual assessments following (Jung et al. 2020). The categories were originally modified from systems for rice (IRRI 1996) and for tropical tree seedlings (Engelbrecht and Kursar 2003)

| **Drought category** | **Visual characteristics** |
| --- | --- |
| **1** | No signs of water stress |
| **2** | Slight signs of wilting without leaf necrosis  (leaf folding, rolling) |
| **3** | Strong signs of wilting without leaf necrosis  (leaf folding, rolling, shrinking, leaf angle change) |
| **4** | Slight leaf tip drying |
| **5** | Tip drying (extended to ¼ length) in 25% of leaves  (normally old leaves) |
| **6** | Tip drying (extended to ¼ length) in 50% of leaves  (or 25% of leaves fully dried) |
| **7** | More than 50% of leaves fully dried |
| **8** | More than 70% of leaves fully dried |
| **9** | All aboveground parts dead |

**Table S5 Size and direction of nutrient effects on drought responses** **(D_surv_, D_resist_, D_legacy_) were not explained by** **a) specific leaf area** (SLA, as rough proxy for resource use strategy, **b) root mass ratio** (RMR, for root biomass allocation), **c) F-value** (for habitat moisture association) or **d) N-value** (for habitat nutrient association, Ellenberg et al. 1992). They were also not explained by **e) nutrient limitation** (assessed as growth rate difference between fertilized and unfertilized plants for each nutrient and moisture treatments separately). In models a)-d) effects of one respective trait and nutrient condition (unfertilized, N, P, NP) including possible interactions on drought responses (D_surv,_ D_resist,_ D_legacy_) across species were analyzed. All models included species’ identity as random effect. D_legacy_ was log transformed to improve model assumptions. In e) effects of nutrient limitation (ΔGR between fertilized and unfertilized plants) on the *change* of each drought response (ΔD_surv_, ΔD_resist_, ΔD_legacy_) with each nutrient addition (N, P, NP) under drought and irrigated conditions were modelled. P_adj_ gives adjusted p-values for multiple testing for drought and irrigated conditions using the holm method. r indicates that Pearson correlation was used to assess relation of nutrient limitation and change in drought performance, correlation coefficient is stated

|  | | **Survival** | | | **drought period** | | | **post-drought period** | | |
| --- | --- | --- | --- | --- | --- | --- | --- | --- | --- | --- |
|  |  | **D_surv_** | | | **D_resist_** | | | **D_legacy_** | | |
| **a) resource use strategy** | df | *χ^2^* | *P* |  | *χ^2^* | *P* |  | *χ^2^* | *P* |  |
| **SLA** | 1 | 1.65 | 0.20 |  | *3.34* | *0.07* | ↓ | 0.75 | 0.39 |  |
| Nutrient condition | 3 | 1.84 | 0.61 |  | 2.87 | 0.41 |  | 5.49 | 0.14 |  |
| SLA x Nutrient condition | 3 | 1.41 | 0.70 |  | 1.41 | 0.70 |  | 2.15 | 0.54 |  |
| Random effects | |  | | |  | | |  | | |
| (1\| species) | | * | | | * | | | * | | |
| R^2^marginal | | 0.07 | | | 0.13 | | | 0.09 | | |
| R^2^conditional | | 0.54 | | | 0.41 | | | 0.51 | | |
| transformation | |  |  |  |  | | | log(D_legacy_+1) | | |

| **b) root biomass allocation** | | df | *χ^2^* | *P* |  | *χ^2^* | *P* |  | *χ^2^* | *P* |  |
| --- | --- | --- | --- | --- | --- | --- | --- | --- | --- | --- | --- |
| **RMR** | | 1 | 0.86 | 0.35 |  | 0.20 | 0.65 |  | 0.04 | 0.84 |  |
| Nutrient condition | | 3 | 1.38 | 0.71 |  | 2.90 | 0.41 |  | 6.10 | 0.11 |  |
| RMR x Nutrient condition | | 3 | 4.56 | 0.21 |  | *6.72* | *0.08* |  | 0.94 | 0.82 |  |
| Random effects | | |  | | |  | | |  | | |
| (1\| species) | | | * | | | * | | | * | | |
| R^2^marginal | | | 0.09 | | | 0.10 | | | 0.07 | | |
| R^2^conditional | | | 0.55 | | | 0.50 | | | 0.51 | | |
| transformation | | |  |  |  |  | | | log(D_legacy_+1) | | |
| **c) habitat moisture association** | | df | *χ^2^* | *P* |  | *χ^2^* | *P* |  | *χ^2^* | *P* |  |
| **F-value** | | 1 | 0.08 | 0.77 |  | 0.99 | 0.32 |  | 0.31 | 0.58 |  |
| Nutrient condition | | 3 | 1.17 | 0.76 |  | 1.74 | 0.63 |  | 6.17 | 0.10 |  |
| F-value x Nutrient condition | | 3 | 5.46 | 0.14 |  | 2.73 | 0.44 |  | 0.10 | 0.99 |  |
| Random effects | | |  | | |  | | |  | | |
| (1\| species) | | | * | | | * | | | * | | |
| R^2^marginal | | | 0.07 | | | 0.10 | | | 0.09 | | |
| R^2^conditional | | | 0.58 | | | 0.46 | | | 0.50 | | |
| transformation | | |  |  |  |  | | | log(D_legacy_+1) | | |
| **d) habitat nutrient association** | | df | *χ^2^* | *P* |  | *χ^2^* | *P* |  | *χ^2^* | *P* |  |
| **N-value** | | 1 | 0.01 | 0.92 |  | 0.24 | 0.62 |  | 0.34 | 0.56 |  |
| Nutrient condition | | 3 | 1.80 | 0.61 |  | 3.12 | 0.37 |  | *7.48* | *0.06* |  |
| N-value x Nutrient condition | | 3 | 2.05 | 0.56 |  | 5.10 | 0.16 |  | 0.50 | 0.92 |  |
| Random effects | | |  | | |  | | |  | | |
| (1\| species) | | | * | | | * | | | * | | |
| R^2^marginal | | | 0.04 | | | 0.11 | | | 0.1 | | |
| R^2^conditional | | | 0.53 | | | 0.42 | | | 0.49 | | |
| transformation | | |  |  |  |  | | |  | | |
| **e) Nutrient limitation ∆GR** | | | **Survival** | | | **drought period** | | | **post-drought period** | | |
|  |  |  | **∆Dsurv** | | | **∆Dresist** | | | **∆Dlegacy** | | |
| **Moisture treatment** | **Nutrient treatment** |  | F | *P* | *P_adj_* | F | *P* | *P_adj_* | F | *P* | *P_adj_* |
| irrigation | N |  | 1.4 | 0.26 | 0.52 | 1.91 | 0.19 | 0.39 | *6.63* | *0.03* | *0.052* |
| drought | N |  | 0.9 | 0.36 | 0.52 | 1.42 | 0.26 | 0.39 | 0.13 | 0.73 | 0.73 |
| irrigation | P |  | 0.3 | 0.6 | 1.0 | r=-0.35 | 0.24 | 0.24 | 1.96 | 0.19 | 0.38 |
| drought | P |  | 0.08 | 0.78 | 1.0 | *3.78* | *0.08* | 0.16 | 0.17 | 0.69 | 0.69 |
| irrigation | NP |  | r=-0.09 | 0.78 | 0.78 | 1.66 | 0.22 | 0.45 | 0.72 | 0.42 | 0.83 |
| drought | NP |  | 1.4 | 0.26 | 0.53 | 0.15 | 0.71 | 0.71 | 0.58 | 0.46 | 0.83 |

# **Methods S2– Supplementary information: Material and Methods**

## Study site, study species and plant material

The study region is characterized by monthly mean temperatures between -1°C and 17.6°C, with July being the warmest month. Monthly precipitation ranges between 46 mm and 92 mm, and rainfall occurs mainly in the growing season from May to September (data from EBG, 1981-2010). The extremely hot and dry summer in 2018 in Central Europe (WMO 2019) was reflected in the local conditions during the experiment (compare Table S1).

All 13 study species (Table 1) are abundant and frequent in extensively managed grasslands in Germany (Socher et al. 2012) and cover a broad range of moisture and nutrient associations (Ellenberg et al. 1992). All species are perennial and have C3 photosynthesis.

Seeds purchased from commercial seed suppliers (Rieger-Hofmann GmbH and Saaten Zeller, Germany) were germinated in seedling trays on quartz sand. They were grown in the greenhouse for four months (Feb‒May 2017) before transplantation to small pots (9 x 5 x 5 cm) in June to allow for root establishment. For transplantation to the common garden experiment at the end of June 2017, we selected similar-sized individuals within each species to minimize intraspecific size variability.

## Experimental design

Plants were transplanted to large boxes with one individual of each species per box, in a rectangular 20 x 20 cm grid to minimize plant-plant interactions, with species assigned randomly to the positions. Plants were exposed to four different nutrient conditions (control (C), N, P, and combined NP, for details,see below) and after a 12 month establishment period additionally to two moisture treatments (drought, irrigated) for eight weeks (drought period), followed by a nine month post-drought period (all irrigated). The boxes were located under transparent rain-out shelters during the moisture treatments, with 9 boxes for each nutrient x moisture treatment (compare main text). For experimental setup, see Figure S1, and for timeline of the experiment see Figure S2. Non-target plants were weeded regularly.

The soil substrate consisted of 60 % sieved sandy topsoil, 30 % quartz sand and 10 % clay (custom soil mixture from Neuland Hum, Erdenwerk Rommel GmbH & Co.KG). It was chosen for relatively low base nutrient concentrations, to minimize nutrient leaching but allow for good drainage, and to mimic natural growing conditions. For drainage, several holes were drilled in the bottom of the boxes, and it was filled with a 10 cm layer of gravel, covered with a layer of filter-fleece (Kgeo 110 TF white; 110g m^-2^).

## Nutrient treatments and conditions in the experiment

The nitrogen addition (N) received the equivalent to 100 kg N ha^-1^yr^-1^ as CH_4_N_2_O, the phosphorus addition (P) the equivalent of 50 kg P ha^-1^yr^-1^as NaH_2_PO_4_*2H_2_O, and the NP addition received a combination of both. The unfertilized control received neither N nor P. The yearly amount of fertilizer was applied as solution divided into three fertilization events per year, at the start of the experiment (2017), before the drought period (2018) and the beginning of the following growing season (2019, see Fig S2). To avoid possible co-limitation, all nutrient treatments including the unfertilized control additionally received the equivalent of 150 kg K ha^-1^yr^-1^as K_2_SO_4_ (with the fertilizations above) and micronutrients (slow-release fertilizer, Micromax Premium, Everris International BV, 100 g worked into the top 5 cm of the soil at the beginning of each growing season).

To evaluate if fertilization treatments affected leaf nutrient contents and plant available soil nutrient contents, we analyzed N and P contents (for methods, see below) before and after the drought period for leaves (usually 3 individuals of each species in each nutrient – moisture treatment combination) and soils (upper 10 cm; 6 boxes per nutrient – moisture treatment combination). Soil samples were taken shortly before the drought treatment started and after the drought treatment, i.e., 3 and 6.5 weeks after the last nutrient application, respectively. Leaf samples were taken at the harvest in May (4-5 weeks after the previous fertilization event) and at the harvest after the drought period (11-17 weeks after the last nutrient application). Nutrient additions increased the leaf content of the respective nutrient (p<0.001) before the drought period (Table S2). At the end of the drought period, nutrient effects either remained or interacted with the drought treatment. Effects on soil nutrient contents were less pronounced, presumably due to depletion through plant nutrient uptake and possibly leaching. For details see, Table S2 and Table S3.

## Irrigation, moisture treatments and moisture conditions during the drought period

Irrigation was implemented with a drip-irrigation system and was adjusted individually for each block and according to weather conditions to ensure non-limiting moisture conditions, i.e., to avoid both soil drying and waterlogging. All boxes were irrigated during the establishment and post-drought period. In the drought period, two moisture treatments were implemented, i.e., in the drought treatment irrigation was discontinued (in half of the boxes) for 56 days leading to a decrease of soil water status through evapotranspiration (Fig S2)., while the other half remained irrigated (irrigation treatment).

All boxes were covered with rainout-shelters from May to September 2018 to protect them from natural precipitation and expose both treatments to the same light and temperature conditions. The rainout shelters (3 m × 3.5 m in size, 2.1 m at the highest point)roofed two boxes each (compare Fig. S1) and were covered with transparent plastic foil (200 μm; Gewächshausfolie UV5, folitec Agrarfolien-Vertriebs GmbH, Westerburg, Germany). To allow for air circulation, two sides of the shelters were entirely open, and the remaining sides were covered down to 50 cm above the ground.

Mean air temperature and mean relative humidity did not differ between moisture treatments (overall 21.9 °C; t-test: p=0.27, and overall, 66.3 %; t-test: p=0.34, respectively). They were assessed with i-buttons (DS1920, Maxim Integrated, CA) 20 cm below the roof of six of the rainout shelters (3 in drought and 3 irrigation treatment).

Soil moisture was monitored in the upper 20 cm during the drought period (for methods, see below). All nutrient conditions reached extremely low soil water potentials of -3.3 MPa at the end of the 56 days of drought treatment (Fig. S3). Nevertheless, the soil in the P-addition dried out slower than the soil in the NP-addition.

## Assessment of plant performance: growth and survival

To assess plant growth, we harvested the aboveground biomass of the plants individually (i.e., cut 6 cm aboveground, mimicking mowing in grasslands) before and after the drought period (May and end of August/ beginning of September 2018 respectively, see timeline Fig. S2), and again at the beginning of the following growing season (May 2019, end of post-drought period). To avoid treatment bias, harvests were conducted block by block and in the same order of boxes. Tillers were harvested together with the mother plant, and tissue that had regrown after re-irrigation (distinguishable by color and texture) was excluded from biomass assessments for growth during the drought period. Biomass was dried at 65°C for at least 72 hours before determining dry weight. Growth rates of the plants under drought and irrigated conditions during the drought period and during the post-drought period were calculated as the biomass at the end of the respective interval, divided by the number of days between harvests (specified for each box).

Assessments and analyses of survival focused on the whole-plant level, i.e., we accounted for potential resprouting from belowground meristems. To that end, whole-plant survival was assessed in May 2019 (after an assessment of aboveground survival in September 2018). It was directly assessed in all 9 boxes in the N and P treatment, and for a subsample of four boxes in each moisture treatment for the unfertilized control and NP treatment. For the remaining boxes (harvested in 2018 for another experiment) it was extrapolated using resprouting probabilities based on the number of individuals that resprouted relative to the number of individuals with no living tissue at the end of the drought period. For calculation of performance parameters and drought responses see main text.

## Testing for associations of parameters with variation of nutrient effects on drought responses

We assessed if species variation of species drought responses (D_surv_, D_resist_, D_legacy_) with nutrient addition is related to their Ellenberg indicator values for moisture and nitrogen (Ellenberg et al. 1992), specific leaf area (SLA) on irrigated plants or root mass ratio (RMR) under full resource availability (irrigated, NP). Using linear regression, we tested the effects of nutrient conditions (N, P, NP) and one trait (habitat association, SLA, RMR), including possible interactions on drought responses across species, separately for each trait. All models included species as random effect to improve model assumptions.

We also assessed if variation of species drought responses with nutrient addition is associated with nutrient limitation (assessed as the difference between fertilized and unfertilized plants ΔGR; separately for each nutrient addition, N, P, NP under each moisture treatment). We used linear regressions to test the relation of the change of each drought response (ΔD_surv_, ΔD_resist_, ΔD_legacy_) with each nutrient addition (N, P, NP) to the strength of nutrient limitation (ΔGR) by the respective nutrient. Models were run separately for each parameter and nutrient and were adjusted for multiple testing. Two models did not fulfill model assumptions even after data transformation (ΔD_surv_ explained by NP-limitation under irrigated conditions and ΔD_resist_ explained by P-limitation under irrigated conditions), and we therefore used Pearson correlation to assess the relation to nutrient-limitation in those cases. Species drought responses were not significantly related to nutrient limitation (Table S5).

## Methods for analyses of leaf and soil nutrient contents

Mass-based leaf N contents were determined with an EA-IRMS coupling (Elemental Analyzer NA 1108, CE Instruments, Milan, Italy). Leaf P contents were determined with inductively coupled plasma optical-emission spectrometry (ICP-OES; Varian Vista-Pro Radial element analyser, Varian Inc., Palo Alto, USA) after HNO3 pressure digestion (Seif digestion, 170°C).

Plant available soil nutrient contents were measured on CaCl_2_ extracts of dried soil samples (using 0.0125 mol CaCl_2_). P concentration was determined with ICP-OES andsoil NH4 and NO3 via Flow-Injection-analysis (FIA;MLE Dresden, FIA-LAB) and ion chromatography (IC, Metrohm), respectively.

## Methods for soil water status analyses

Soil moisture was monitored with a combination of methods, due to different measuring ranges and effort: *Volumetric* soil water content was initially followed in both moisture treatments twice per week (time domain reflectometry, Hydrosense II; Campbell Scientific, Inc. Logan, Utah, USA). However, in the drought treatment the soil soon got too hard to insert the sensor into the soil (day 19 of drought). We then assessed *gravimetric* soil water content (on soil cores 10-20 cm depth) in three boxes per nutrient condition in both moisture treatments three times (day 22, 26 and 45 of the drought period). Additionally, in the drought treatment we directly assessed *soil water potentials* twice per week (at 20 cm depth in 15 boxes, from day 16 of drought) using soil psychrometers (Merrill Specialty Equipment, Logan, Utah, USA; and PSYPRO™; water potential system, Wescor, Inc., Logan, Utah, USA). These *psychrometric* measurements were taken until they reached values below the measurement range of -10MPa (week 5). Volumetric and gravimetric soil water contents were converted to soil water potentials based on a water retention curve of the soil in the experiment (WP4C Dewpoint Potentia Meter, METER Group, Munich, Germany). **References**

EBG Monthly precipitation and temperature years 1981-2010; 2018, Ecological Botanical Garden, University of Bayreuth. https://www.bayceer.uni-bayreuth.de/meteo/en/klimastati/gru/html.php?id_obj=140009

Ellenberg H, Weber HE, Düll R, Wirth V, Werner W, Paulißen D (1992) Zeigerwerte von Pflanzen in Mitteleuropa. 2. und verbesserte Auflage. Scr Geobot 18:1–258

Engelbrecht BMJ, Kursar TA (2003) Comparative drought-resistance of seedlings of 28 species of co-occurring tropical woody plants. Oecologia 136:383–393. https://doi.org/10.1007/s00442-003-1290-8

IRRI (1996) Standard evaluation system for rice. Int Rice Res Institute, Los Baños

Jung E-Y, Gaviria J, Sun S, Engelbrecht BMJ (2020) Comparative drought resistance of temperate grassland species: testing performance trade-offs and the relation to distribution. Oecologia 192:1023–1036. https://doi.org/10.1007/s00442-020-04625-9

Socher SA, Prati D, Boch S, Müller J, Klaus VH, Hölzel N, et al (2012) Direct and productivity-mediated indirect effects of fertilization, mowing and grazing on grassland species richness. J Ecol 100:1391–1399. https://doi.org/10.1111/j.1365-2745.2012.02020.x

WMO (2019) WMO Statement on the State of the Global Climate in 2018, WMO-No. 1233. World Meteorological Organization (WMO‐No. 1233) Geneva, Switzerland, Geneva, Switzerland
